# *De Novo* Transcriptome Sequence Assembly from Coconut Leaves and Seeds with a Focus on Factors Involved in RNA-Directed DNA Methylation

**DOI:** 10.1534/g3.114.013409

**Published:** 2014-09-04

**Authors:** Ya-Yi Huang, Chueh-Pai Lee, Jason L. Fu, Bill Chia-Han Chang, Antonius J. M. Matzke, Marjori Matzke

**Affiliations:** * Institute of Plant and Microbial Biology, Academia Sinica, Taipei, Taiwan; † Yourgene Bioscience, Taipei, Taiwan

**Keywords:** coconut, endosperm, epigenetics, monocot, RNA-seq

## Abstract

Coconut palm (*Cocos nucifera*) is a symbol of the tropics and a source of numerous edible and nonedible products of economic value. Despite its nutritional and industrial significance, coconut remains under-represented in public repositories for genomic and transcriptomic data. We report *de novo* transcript assembly from RNA-seq data and analysis of gene expression in seed tissues (embryo and endosperm) and leaves of a dwarf coconut variety. Assembly of 10 GB sequencing data for each tissue resulted in 58,211 total unigenes in embryo, 61,152 in endosperm, and 33,446 in leaf. Within each unigene pool, 24,857 could be annotated in embryo, 29,731 could be annotated in endosperm, and 26,064 could be annotated in leaf. A KEGG analysis identified 138, 138, and 139 pathways, respectively, in transcriptomes of embryo, endosperm, and leaf tissues. Given the extraordinarily large size of coconut seeds and the importance of small RNA-mediated epigenetic regulation during seed development in model plants, we used homology searches to identify putative homologs of factors required for RNA-directed DNA methylation in coconut. The findings suggest that RNA-directed DNA methylation is important during coconut seed development, particularly in maturing endosperm. This dataset will expand the genomics resources available for coconut and provide a foundation for more detailed analyses that may assist molecular breeding strategies aimed at improving this major tropical crop.

The coconut palm (*Cocos nucifera* L., Arecaceae) is one of the most important crops in tropical zones, and it plays a significant role in the economy and culture in many tropical countries (Gunn *et al.* 2011). Coconut is the only species within the genus *Cocos* and it is morphologically classified into two types: tall and dwarf. Tall coconuts are outbreeding, whereas the dwarf variety, which is thought to result from human selection, is mainly self-pollinating (Gunn *et al.* 2011). Despite the importance of coconut for humans and tropical ecosystems, available genetic sequences are relatively scarce as compared with other economically important palms, oil palm and date palm, for which whole genome sequences and transcriptome data are available (Yang *et al.* 2010; Al-Dous *et al.* 2011; Bourgis *et al.* 2011; Fang *et al.* 2012; Uthaipaisanwong *et al.* 2012; Yin *et al.* 2012; Zhang *et al.* 2012; Al-Mssallem *et al.* 2013; Dussert *et al.* 2013; Singh *et al.* 2013). In the case of coconut, much of the recent effort has been limited to marker development to assist with cultivar identification (Perera *et al.* 2000, 2003; Perera *et al.* 2008; Gunn *et al.* 2011). Molecular studies at a genome-wide scale using next-generation sequencing are even scarcer. The sequence of the coconut chloroplast genome was recently determined (Huang *et al.* 2013). However, a whole genome sequence has not yet been reported and the only published genome-wide study of coconut is a transcriptome analysis of a tall variety using combined tissues of leaves and fruit flesh (Fan *et al.* 2013). Additional work is thus needed to increase coconut genomic and transcriptomic resources, which may provide new insights for the discovery of novel genes linked to important agronomic traits.

In addition to being a prominent tropical crop, coconut is also interesting to investigate from the perspective of comparative seed development. Flowering plants can be divided into two major groups. Coconut, rice, wheat, barley, and maize are examples of monocotyledonous plants (monocots), whereas *Arabidopsis thaliana* (Arabidopsis) and legumes are representatives of the dicotyledonous group (dicots). Double fertilization in both groups produces seeds containing a diploid embryo and a triploid endosperm, which acts as a nutrient store for the developing or germinating embryo. In some monocots, including cereals and coconut, the endosperm persists in the mature seed and supplies an important source of nutrition in the human diet. Coconut has the second largest seed in the world, exceeded only by *Lodoicea maldivica*, another palm species endemic to the Seychelles. Seed size, which is a determinant of crop yield, is dependent on many factors, including maternal influences, epigenetic processes, and endosperm growth (Sreenivasulu and Wobus 2013). Similar to other monocots and dicot plants with albuminous seeds such as coffee and castor bean (Joët *et al.* 2009), the embryo in coconut is tiny compared with the abundant endosperm. In late developing and mature coconut seeds, the endosperm, which supplies the major edible portion of coconut, can be 100-times the weight of the corresponding embryo (Supporting Information, Figure S1).

Epigenetic processes, including those involving small RNAs and components of the RNA-directed DNA methylation (RdDM) machinery (Matzke and Mosher 2014), have important roles during seed development. Epigenetic alterations of chromatin, which include not only DNA methylation but also various histone modifications, are necessary to regulate seed-specific genes and to protect genome integrity by silencing transposons. In addition, epigenetic modifications are required in the endosperm to establish parental imprinting (Gehring 2013), an epigenetic phenomenon of allele-specific expression that can influence seed size (Sreenivasulu and Wobus 2013). Embryo and endosperm transcriptomes have been determined for several other plant species, including Arabidopsis (Belmonte *et al.* 2013) and rice (Xu *et al.*, 2012; Gao *et al.* 2013). Comparative transcriptomes of fruit, seed, and mesocarp tissues with an emphasis on fatty acid composition and metabolism at different developmental stages were determined for oil palm and date palm (Bourgis *et al.* 2011; Dussert *et al.* 2013). However, detailed transcriptome studies during seed development have not yet been extended to many nonmodel plants. Coconut represents an interesting nonmodel plant that produces exceptionally large seeds containing copious amounts of endosperm at later stages of development and small but macroscopically visible embryos. Coconut seeds thus provide an opportunity to investigate the expression of epigenetic factors in a developmental context that is unique.

With these considerations in mind, we have performed *de novo* transcriptome assembly of RNA-seq libraries prepared from seed tissues (mature embryos and gelatinous endosperm) and leaves of a dwarf coconut plant. We report the findings from our analysis of the transcriptome data from these three tissues with a focus on identification of putative homologs of factors required for RdDM.

## Materials and Methods

### Plant materials and RNA extraction

For this study, we used a dwarf variety of coconut provided by Mr. Chi-Tai Lin, a private breeder who imported the first batch of green dwarf coconuts with strong taro fragrance to Taiwan from Thailand approximately 30 yr ago and started to breed this particular variety in Hengchun peninsula, Pingtung County, southern Taiwan. RNA-Seq data were collected from maturing gelatinous endosperm (coconut approximately 5 months old, embryo invisible at this stage), nearly mature embryo (coconut at approximately 8 months old), and young leaf (seedling at approximately 8 months old) (Figure S1). Total RNA from each tissue was extracted using a Plant Total RNA Miniprep Purification Kit (GMbiolab Co, Ltd., Taichung, Taiwan). We used the ratio of absorbance at 260 nm and 280 nm (A260/280) and gel electrophoresis to measure the purity and integrity of the extracted RNA. The mRNA molecules were isolated from high-quality RNA (concentration >300 ng/µl; A260/230 > 1.7; A260/280 = 1.8–2.1).

### Library construction and sequencing

We used TruSeq RNA Sample Preparation kit for cDNA library construction (Illumina, San Diego, CA). First, poly-T oligo-attached magnetic beads were used to isolate mRNA from total RNA. The isolated mRNA was fragmented and reverse-transcribed to single-stranded cDNA with random primers, forming a mixture of DNA/RNA hybrid. The second-stranded cDNA was then synthesized and purified. During the synthesis of the second stranded cDNA, deoxythymidine triphosphate (dTTP) was substituted with deoxyuridine triphosphate (dUTP), which helped enforce strand specificity (Wang *et al.* 2011). The resulting double-stranded cDNA was end-repaired and adenylated, followed by poly A tailing and paired-end Y-adaptor ligation, and then treated with uracil-DNA glucosylase (UDG) to digest the dUTP-marked strand. The selected strand was amplified and screened through agarose gel electrophoresis. Fragments ranging from 350 bp to 520 bp were selected for later sequencing. Paired-end sequencing using IlluminaHiSequation 2000/2500 was executed at YourGene Bioscience Co. (New Taipei City, Taiwan) with a maximum read length of 201 and a minimum read length of 101. All raw reads were deposited in the Sequence Read Archive (SRA) at NCBI under the accession number SRP041201.

### *De novo* assembly, gene annotation, and expression, GO, and KEGG annotation

Bases of raw reads resulting from Illumina sequencing were trimmed with an error probability of 0.05 using CLC Genomic Workbench 6.0.1 (CLC bio, Aarhus, Denmark). Trimmed reads shorter than 35 bp were filtered out. Clean reads were put into Velvet 1.2.07 (Zerbino and Birney 2008) for initial assembly. Five k-mer values (35, 45, 55, 65, and 75) were set for our preliminary assembly. The resulting contigs were then merged for later procedure. Final contigs produced from Velvet were uploaded to Oases 0.2.06 (Schulz *et al.* 2012) for transcript assembly and isoforms construction. Transcript isoforms of a locus with highest confidence score were selected as unigene for that locus. If two isoforms of the same locus have the same confidence score, then the one with longer length was selected as unigene. Unigene sequences were deposited in the Transcriptome Shotgun Assembly (TSA) Sequence Database at NCBI under the accession numbers of GBGL00000000 for embryo, GBGK00000000 for endosperm, and GBGM00000000 for leaf transcriptomes. To evaluate the expression of unigenes, we first mapped trimmed reads to unigene sequence using gapped alignment mode of the program Bowtie 2.2.1.0 (Langmead *et al.* 2009). After alignment, we quantified gene expression with the software package eXpress 1.3.0 (Roberts and Pachter 2013), which reported the abundances of unigenes in the form of the fragments per kilobase of transcript per million mapped reads (FPKM). The identity of the unigene was annotated by BLAST (blastx) search against nonredundant (nr) protein database maintained by NCBI with an E-value cutoff of 10^−5^. The nr protein database is a combination of SwissProt, SwissProt updates, the Protein Identification Resource (PIR), and the Protein Data Bank (PDB).

To determine the similarity of our transcriptome data with previous transcriptome data from palms, we performed BLAST search against available EST sequences of *Cocos nucifera* (1005 sequences), *Elaeis guineensis* (40,920 sequences), and *Phoenix dactylifera* (411 sequences) downloaded from GenBank. Moreover, we also aligned our unigenes against transcripts of fruit and seed tissues of oil palm (minimum 20 reads per million; 20,077 sequences in total) (Dussert *et al.* 2013).

To obtain the Gene Ontology (GO) annotation and perform enrichment analysis, accession numbers acquired from BLAST search were queried in the GO database using BLAST2GO 2.6.4 (Conesa *et al.* 2005; Gotz *et al.* 2008). For analysis of individual GO category enrichment, the reference dataset was obtained from the summation of unigenes matching to the same accession numbers. The significance of the enrichment was evaluated by Fisher’s exact test. To understand high-level functions and utilities of the biological systems, we also performed the KEGG (Kyoto Encyclopedia of Genes and Genomes) pathway annotation as described by Kanehisa *et al.* (2012).

### Factors involved in RNA-directed DNA methylation

Factors in involved in RNA-directed DNA methylation (RdDM) were manually selected from annotated unigene pools. The sequences indicated in Table 3 were validated by Sanger sequencing and submitted to GenBank. They can be accessed through the following accession numbers: KJ851186–KJ851206. Real-time PCR (qPCR) was used for transcription profiling of four key RdDM-related genes (DRM, NRPD1, NRPE1, and MET1) in three and two different developmental stages of endosperm and embryo, respectively. The three stages of endosperm we used are gelatinous, early white solid (thin layer), and late white solid (thick layer) endosperms. The two embryos were collected from the same inflorescence that was used for collections of late white solid endosperm. Primers were designed based on assembled sequences and a complete primer list is provided in Table S1.

## Results and Discussion

### Sequencing and *de novo* assembly

A summary of the sequencing results and *de novo* assembly is presented in Table 1. A total of 10 GB raw sequence was obtained for each tissue. Of the three tissues, the leaf transcriptome has the maximum total number of sequencing reads (121,151,552) with an average read length of 126 bp and an inserted size of 286 bp, whereas the embryo transcriptome has the minimum total sequencing reads (81,128,552) with an average read length of 168.5 bp. After quality trimming, the average read lengths are 158.3 bp, 97.9 bp, and 120.36 bp in transcriptomes of embryo, endosperm, and leaf, with an inserted size of 430 bp, 306 bp, and 286 bp, respectively. The *de novo* assembly of clean reads produced the most total transcripts in endosperm (229,866) and the minimum in embryo (86,254). Total unigenes in the former are 61,125 with an average length of 684 bp and a GC content of 41%, whereas the latter have 58,211 total unigenes with an average length of 732 bp and a GC content of 48%. Interestingly, although leaf transcriptome has more total transcripts (159,509) than embryo, its total unigenes (33,446) are far less than embryo transcriptome. However, its unigenes have the longest average length (744 bp) and highest GC content (48%). The length distribution of total unigenes found in three tissues is shown in Figure S2.

**Table 1:** Sequencing results and *de novo* assembly

Sequencing Results and *De Novo* Assembly	Embryo	Endosperm	Leaf
Sequencing results			
Total Illumina reads (No)	81,128,552	103,080,366	121,151,552
Average read length (bp)	168.5	101	126
Total base (No)	13,670,161,012	10,411,116,966	15,265,095,552
Total reads after QT (No)	78,553,435	99,253,456	120,061,360
Average read length after QT (bp)	158.3	97.9	120.36
Total clean base (No)	12,435,008,760	9,682,020,834	13,609,335,965
Insert size (bp)	430	306	286
*De novo* assembly			
Total transcripts (No)	86,254	229,866	159,509
Total unigenes (No)	58,211	61,152	33,446
Average contig length of unigene (bp)	732	684	744
Unigenes with multiple hits (No)	24,857	29,731	26,064
Unigenes with unique hits (No)	23,836	22,278	20,844
N50	951	969	912
GC content of unigene (%)	41	45	48

### Gene expression and annotation

**Figure 1:**
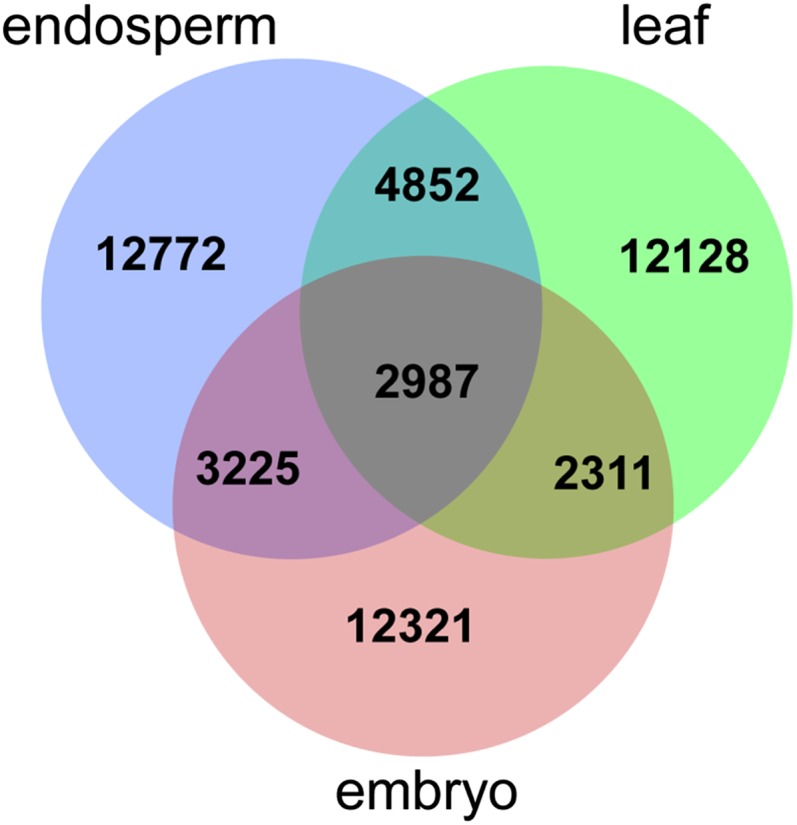
Venn chart showing unique and shared unigenes found in three coconut transcriptomes.

Our blast search of unigenes against the nr protein database in NCBI with an E-value cutoff of 10^−5^ led to the identification of 24,857 (42.7%), 29,731 (48.6%), and 26,064 (77.9%) annotated unigenes with multiple matches from embryo, endosperm, and leaf transcriptomes, respectively (Table 1). Of the annotated unigenes with a unique match, 2987 were shared by all three tissues, 3225 were shared by endosperm and embryo, 4852 were shared by endosperm and leaf, and 2311 were shared by embryo and leaf (Figure 1). Numbers of tissue-specific unigenes are 12,772 in endosperm, 12,321 in embryo, and 12,128 in leaf (Figure 1).

**Figure 2:**
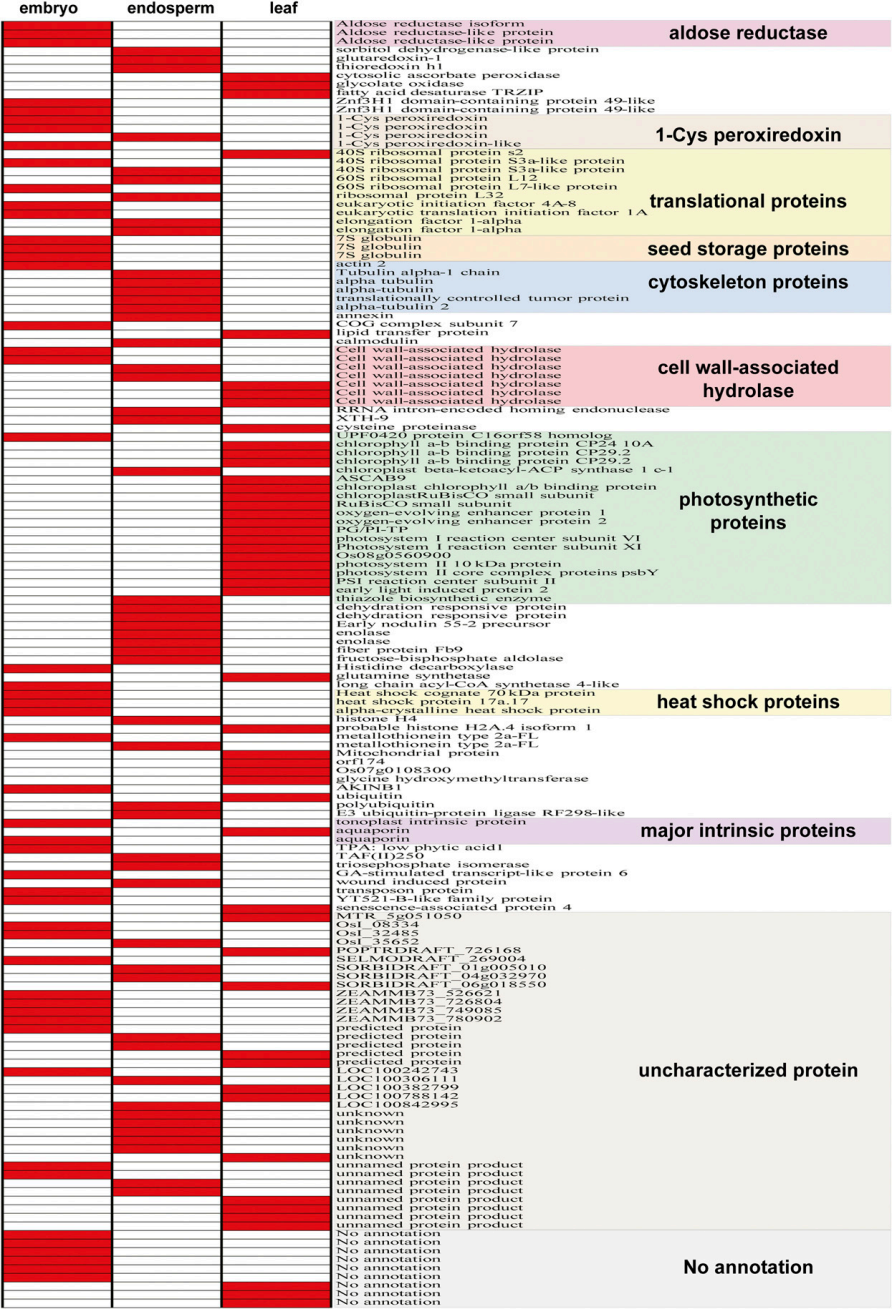
Expression profile of top 50 expressed genes in the three tissues. The colors denote absence (white) and presence (red) of a particular gene transcript. Photosynthetic genes are almost exclusively found in the leaf transcriptome. Seed storage (7S globulin) and heat shock proteins are prominent in the embryo. Translational and cytoskeleton proteins are abundant in embryo and in endosperm, but rarely found in leaf. Cell wall–associated hydrolase and major intrinsic proteins are evenly distributed in three tissues. Uncharacterized proteins exist in all three tissues, but unigenes without matched sequences in GenBank are found only in embryo and in leaf, not in endosperm.

Complete gene expression lists (FPKM > 1) from the embryo, endosperm, and leaf transcriptomes are provided as Table S2, Table S3, and Table S4. Expression profiling of the top 50 expressed genes (Figure 2) reflects the physiological characteristics and/or function of the different tissues. As expected, photosynthetic genes are found almost exclusively in the leaf transcriptome, with the most abundant transcript encoding the small subunit of ribulose-1,5-bisphosphate carboxylase (Table 2). In endosperm, which has been sampled at the gelatinous stage undergoing active cell division (Abraham and Mathew 1963), transcripts for cytoskeletal and translational proteins are abundant. Alpha-tubulin, which forms microtubules in the mitotic spindle, is the most highly transcribed gene in endosperm and translationally controlled tumor protein (Amson *et al.* 2013), which binds to microtubules (Santa Brigida *et al.* 2014) and regulates cell division (Brioudes *et al.* 2010), is in the top 10 (Table 2). Other highly expressed genes in coconut endosperm (Table 2), including annexin, enolase, and metallothionein type 2A (MT2A), are also among those expressed at a high level in endosperm of castor bean (Lu *et al.* 2007) and *Brassica napus* (Huang *et al.* 2009).

**Table 2:** Top expressed genes identified in three tissues of coconut

Embryo		Endosperm		Leaf	
Transcript Annotation	FPKM	Transcript Annotation	FPKM	Transcript Annotation	FPKM
Metallothionein type 2a-FL	12328.2	Alpha-tubulin	11020.0	Chloroplast RubisCO small subunit	71647.2
7S globulin	8069.0	Dehydration responsive protein	7930.5	Os08g0560900	29745.4
Aldose reductase-like protein	2412.7	Unnamed protein product	6377.5	Ubiquitin	17929.8
Long chain acyl-CoA synthetase 4-like	2410.7	Elongation factor 1-alpha	5994.5	Mitochondrial protein	17284.7
Cell wall–associated hydrolase	1910.6	Translationally controlled tumor protein	5012.9	ASCAB9	13504.6
GA-stimulated transcript-like protein 6	1817.0	Cell wall–associated hydrolase	4972.3	Chloroplast chlorophyll a/b binding protein	12838.5
Zn3H1 domain-containing protein 49-like	1391.0	SORBIDRAFT_04g032970	4285.5	Photosystem I reaction center subunit XI	12695.6
1-Cys peroxiredoxin	1322.4	Sorbitol dehydrogenase-like protein	4042.6	No annotation	8909.8
Eukaryotic translation initiation factor 1A-like	1316.9	RRNA intron-encoded homing endonuclease	3847.1	Unknown	8812.3
OsI_08334	1304.3	Ribosomal protein L32	3650.3	Early light-induced protein 2	8496.1
AKIN beta1	1239.4	Polyubiquitin	3581.0	Unnamed protein product	6946.9
Heat shock protein 17a	1179.5	Metallothionein type 2a-FL	3522.5	Oxygen-evolving enhancer protein 2	5528.4
Tonoplast intrinsic protein	1175.2	Early nodulin 55-2 precursor	3063.2	Predicted protein	5031.4
Actin	1089.8	Unknown	2991.1	POPTRDRAFT_726168	5019.5
ZEAMMB73_780902	1013.8	Thiazole biosynthetic enzyme	2620.5	Glycine hydroxymethyltransferase	4332.1
No annotation	974.0	Predicted protein	2568.3	Cell wall–associated hydrolase	4316.6
Aldose reductase isoform 1	963.7	Glutaredoxin-1	2499.4	Lipid transfer protein	4310.7
ZEAMMB73_726804	881.6	Annexin	2453.6	Probable histone H2A.4 isoform 1	4241.2
ZEAMMB73_749085	857.3	SORBIDRAFT_01g005010	2298.0	LOC100788142	4080.4
Histidine decarboxylase	820.8	Thioredoxin h1	2243.8	MTR_5g051050	3984.3
OsI_32485	764.1	1-Cys peroxiredoxin	2138.4	40S ribosomal protein s2	3525.6
Unnamed protein product	736.0	Enolase	2113.7	Senescence-associated protein 4	3431.4
60S ribosomal protein L7-like	693.9	Calmodulin	2091.7	Glycolate oxidase	3425.2

In mature coconut embryos, genes encoding 7S globulin storage protein are among the most highly transcribed (Table 2) because they are in oil palm embryos at later stages of development (Morcillo *et al.* 1997). However, the most highly transcribed gene in embryos encodes MT2A (Table 2). MTs are small cysteine-rich proteins with proposed roles in stress responses and metal storage, transport, and detoxification (Leszczyszyn *et al.* 2013). The first plant MTS were discovered in wheat embryos (Leszczyszyn *et al.* 2013), and our finding of high expression of MT2A in mature coconut embryos is consistent with a crucial but still undefined role late in embryogenesis. High expression of MTs has also been observed in ripening pineapple (Moyle *et al.* 2005) and banana (Liu *et al.* 2002), as well as developing Douglas fir seeds (Chatthai *et al.* 1997) and oil palm embryoids and embryonic callus (Low *et al.* 2008). Uncharacterized proteins exist in all three tissues, but unigenes without matched sequences in GenBank are found only in embryo and leaf, not endosperm (Figure 2).

**Figure 3:**
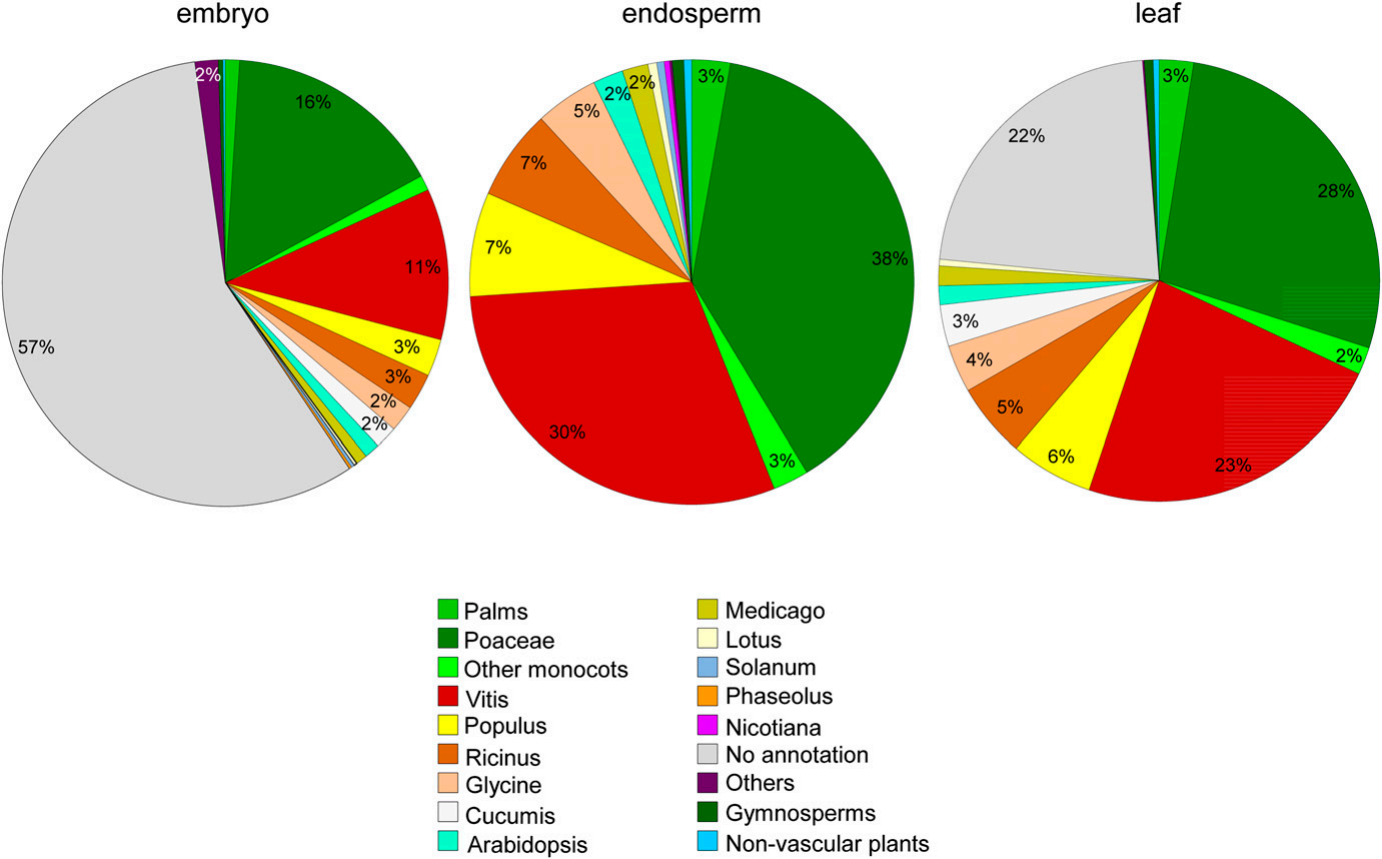
Species distribution of coconut transcripts (FPKM >1.00) resulting from de *novo* assembly. Sections <2% are not labeled.

Our blast search showed that a significant number of unigenes in the embryo transcriptome (57%) and leaf transcriptome (22%) do not have any match in GenBank (Figure 3). Matches between coconut unigenes and entries of nr protein database showed that species of monocots have the best match (highest total score), particularly from the Poaceae (true grass) family, which includes 16%, 38%, and 28% of the best matches in embryo, endosperm, and leaf transcriptomes (Figure 3). Somewhat surprisingly, the species with the second-best match was *Vitis vinifera* (common grape vine), which contains 11%, 30%, and 23% of the best matching sequences in embryo, endosperm, and leaf transcriptomes. The significance of these similarities in protein sequence between two rather distantly related plants (monocot coconut and dicot grape) is not yet clear, but a relatively high percentages of matches to Vitis were also found for other monocots such as date palm (Al-Dous *et al.* 2011), pineapple (Ong *et al.* 2012), and banana (Passos *et al.* 2013). Other eudicots combined together to comprise 10%–25% of the best matches. A small proportion of unigenes matched to members of basal angiosperms, gymnosperms, and ferns. Very few unigenes matched to sequences of nonvascular plants (bryophytes) or green algae.

Although our search against the nr protein database found only few matches to palm sequences (1%–3%) (Figure 3), the search against palm EST sequences (42,336 sequences in total) showed that the matches between our datasets and palm EST sequences are 13,445 (23.10%) for embryo, 15,835 (25.90%) for endosperm, and 14,730 (44.04%) for leaf transcriptomes. However, the BLAST search against 20,077 oil palm transcripts (Dussert *et al.*, 2013) showed that the matches for our embryo, endosperm, and leaf unigenes are 15,906 (27.3%), 16,034 (26.22%), and 16,010 (47.84%), respectively.

### GO annotation

Our GO annotation at level two assigned 88,224, 123,063, and 106,751 GO terms to annotated unigenes with unique hits of embryo (20,844), endosperm (22,278), and leaf (23,836) transcriptomes (Figure 1) under the categories of cellular component, molecular function, and biological process. Averages of three, five, and four GO terms were assigned per unigenes for embryo, endosperm, and leaf transcriptomes, respectively. Similar distribution pattern of GO classification was distinguished among three tissues (Figure S3), with the majority of the GO terms being assigned to biological process distributed in 21 subcategories (∼49%), followed by cellular component in eight subcategories (∼31%) and molecular function in 12 subcategories (∼20%). Of these GO terms, proteins participating in cellular and metabolic processes are the most abundant, counting 23% and 21% of the total GO terms of biological process in three tissues. Of the cellular component, the most dominant proteins are those involved in cell and organelle developments, which count 37% and 29% of the total GO terms of cellular component. Of the molecular function, proteins acting on catalytic activity and binding take up to 43% and 41% of the total GO terms of molecular function. A similar distribution pattern has also been demonstrated in a tall coconut variety by Fan *et al.* (2013), as well as in banana (Passos *et al.* 2013) and pineapple (Ong *et al.* 2012).

**Figure 4:**
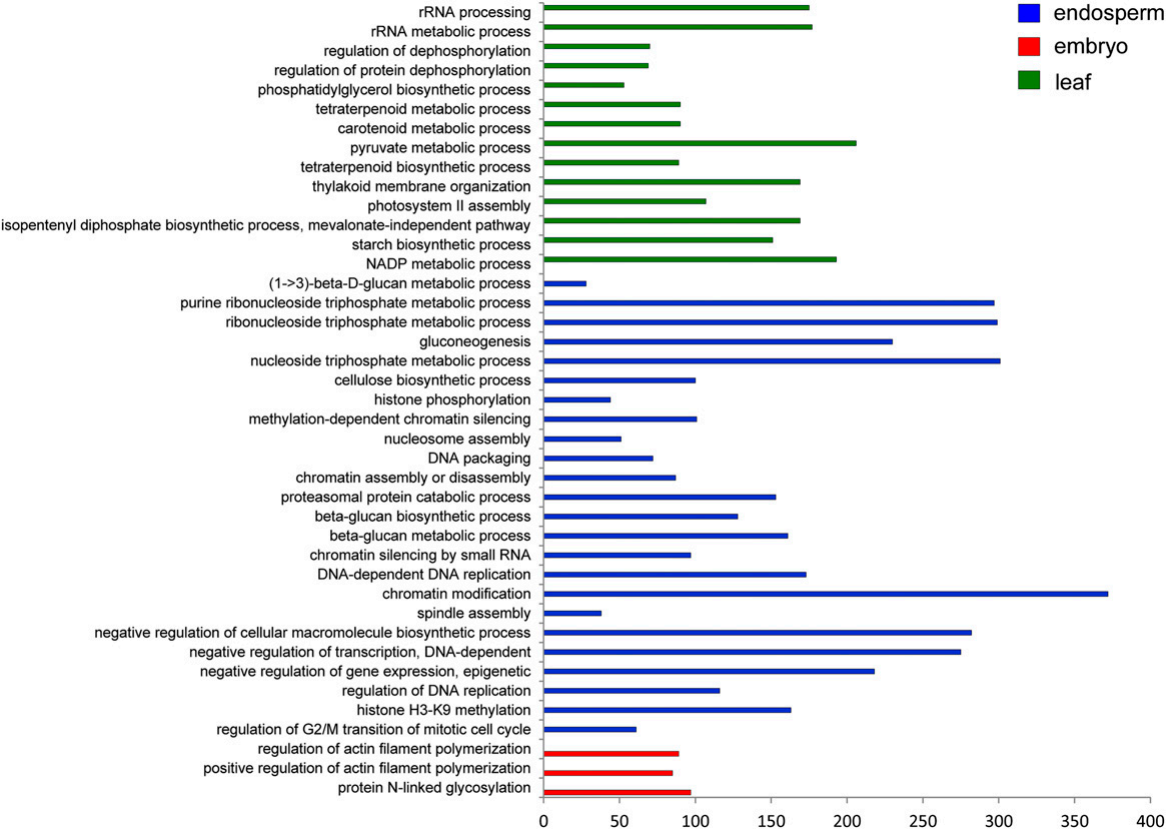
Analysis of GO enrichment at level eight.

We performed further GO analysis at level eight. Details of each main category can be found in the Supporting Information (Figure S4 and Figure S5 and Table S5, Table S6, Table S7). In addition, we assessed the enrichment of annotated GO terms at level eight for the category of biological process. This analysis indicated that three GO terms are significantly enriched (*P* < 0.01) in embryo, 24 are significantly enriched in endosperm, and 14 are significantly enriched in leaf transcriptomes (Figure 4). As anticipated, GO terms associated with photosynthesis and light responses are prominent in the leaf transcriptome. Consistent with gelatinous endosperm comprising actively dividing cells engaged in epigenetic processes, GO terms associated with chromatin modifications and assembly, RNA metabolism, and mitosis are enriched in the endosperm transcriptome (Figure 4).

### KEGG analysis

A KEGG analysis (Kyoto Encyclopedia of Genes and Genomes) was performed to identify active biological pathways in the coconut tissues undergoing investigation. This analysis assigned 5686 unigenes distributing in 138 pathways for embryo transcriptome. There were 7707 unigenes assigned to 138 pathways and 5686 unigenes assigned to 139 pathways for endosperm and leaf transcriptomes, respectively (Table S8). Our results are comparable with a previous RNA-seq transcriptome study of pooled coconut tissues (spear leaves, young leaves, and fruit flesh, Hainan Tall cultivars) that identified 57,304 unigenes, 23,168 of which could be mapped to 215 KEGG pathways (Fan *et al.* 2013). As noted in the previous study (Fan *et al.* 2013), it is interesting to consider genes involved in fatty acid biosynthesis and metabolism. Derived from coconut meat (mature endosperm), coconut oil contains a high proportion of medium chain fatty acids, including commercially important lauric acid (Kumar 2011). In their pooled coconut transcriptomes, Fan *et al.* (2013) found 347 genes involved in the five steps of fatty acid and metabolism (fatty acid biosynthesis, unsaturated fatty acid, citrate cycle, fatty acid metabolism, and fatty acid elongation). We identified 230, 361, and 335 unigenes for embryo, endosperm, and leaf transcriptomes in these pathways (Table S8). Unigenes for fatty acid biosynthesis, elongation, and metabolism were most highly represented in the endosperm transcriptome (Table S8). A comparative transcriptome analysis in three oil palm fruit and seed tissues revealed expression in endosperm of many genes involved in fatty acid synthesis (Dussert *et al.* 2013).

### RdDM factors

RNA interference (RNAi) is an umbrella term describing gene silencing pathways that use Dicers, Argonautes (AGO), and RNA-dependent RNA polymerases (RDR) to make and use small RNAs (20–30 nt in length) that elicit sequence-specific gene silencing (Martínez de Alba *et al.* 2013). Genes encoding these factors have amplified and functionally diversified in plants (Xie *et al.* 2004; Kapoor *et al.* 2008; Liew *et al.* 2013; Nakasugi *et al.* 2013; Liu *et al.* 2014). The Arabidopsis genome encodes four DICER-LIKE (DCL), 10 AGO, and six RDR proteins (Xie *et al.* 2004), whereas rice has eight DCL, 19 AGO, and five RDR genes (Kapoor *et al.* 2008). In the combined data set from the three coconut tissues, we identified at least partial transcripts for four DCL, four AGO, and four RDR proteins (Table 3), including those specialized for RdDM (discussed below). It is likely that additional members of these families remain to be identified in coconut once a whole genome sequence is available and RNA-seq technology is extended to other tissues types, developmental stages, and environmental conditions.

**Table 3:** Putative RdDM factors and related RNAi proteins identified in coconut tissues

Name	Transcript ID	Length		BLASTX best hit organism				Accession
		nucleotide	amino acid	Name and ID	Length (AA)	Coverage (%)	Identity (%)	
RPA1	Endo_Locus_19151	5417	1661	*Oryza sativa* (BAD35511)	1748	95	56	KJ851186
RPB1	Leaf_211	5318	1536	*Oryza brachyantha* (XP_006659128)	1855	86	94	KJ851187
NRPD1	Leaf_Locus_3193	1712	568	*Oryza brachyantha* (XP_006653694)	1386	99	57	KJ851188
NRPE1	Endo_Locus_10009	5244	1672	*Aegilops tauschii* (EMT23234)	2017	95	52	KJ851189
DCL1	Endo_Locus_3719	5490	1534	*Vitis vinifera* (XP_002268369)	1971	93	85	KJ851190
DCL3a	Endo_Locus_25736	3777	1206	*Vitis vinifera* (XP_002280293)	1648	95	61	KJ851191
DCL3b	Endo_Locus_6769	3715	966	*Oryza sativa* (ABB20894)	1116	78	43	KJ851192
DCL4	Embryo_Locus_7881	5576	1617	*Setaria italica* (XM_004976122)	1632	86	63	KJ851193
AGO1	Endo_Locus_673	3410	864	*Vitis vinifera* (XP_002271225)	1085	76	89	KJ851194
AGO2	Embryo_Locus_4735	918	251	*Oryza sativa* (NP_001053871)	1034	82	46	KJ851195
AGO4	Endo_Locus_25417	2258	659	*Vitis vinifera* (XP_002275928)	913	87	77	KJ851196
AGO10	Embryo_Locus_15517	1079	313	*Vitis vinifera* (XP_002279408)	995	31	48	KJ851197
RDR 1	Endo_Locus_54052	406	134	*Vitis vinifera* (XP_002284914)	1121	99	74	KJ851198
RDR 2	Embryo_Locus_43307	2041	616	*Vitis vinifera* (XP_002280099)	1127	90	67	KJ851199
RDR 5	Leaf_Locus_25001	1809	590	*Populus trichocarpa* (XP_006380469)	899	97	60	KJ851200
RDR 6	Endo_Locus_13061	3140	711	*Nicotiana tabacum* (ADI52625)	1197	75	34	KJ851201
MET1	Endo_Locus_26121	4775	1518	*Elaeis guineensis* (ABW96888)	1543	98	97	KJ851202
DRM	Leaf_Locus_2627	2022	554	*Elaeis guineensis* (ABW96890)	591	82	86	KJ851203
CMT	Endo_Locus_4191	2245	603	*Elaeis guineensis* (ABW96889)	925	94	89	KJ851204
ROS1	Endo_Locus_23310	620	108	*Citrus sinensis* (AGU16984)	1573	52	67	KJ851205
DRD1	Endo_Locus_31250	445	93	*Theobroma cacao* (EOX97924) 899	899	62	62	KJ851206

Note: In a search of a list of date palm expressed genes (Supplementary Data 1, Al-Mssallem *et al.* 2013), we could find one RPA1 (KacstDP.mRNA.S000004.203), three AGO proteins (KacstDP.mRNA.S000249.22, KacstDP.mRNA.S001251.1 and KacstDP.mRNA.S000009.162) and one RDR2 (KacstDP.mRNA.S000670.5) proteins. In a BLAST search of oil palm unannotated, *de novo* assembled transcripts (Supplemental Data 1, 2 and 3, Dussert *et al.* 2013), we identified four RPB1 (CL1714Contig3, CLContig342, CL1Contig406 and CL1Contig7380), one NRPE1 (CL1715Contig1), one DCL3b (CL1841Contig3), five AGO1 (CL1Contig1334, CL1Contig4087, CL1Contig7749, CL1Contig2199 and CL1721Contig1), four AGO4 (CL1Contig2911, CL1Contig7017, CL665Contig1 and CL665Contig3), one RDR1 (CL1Contig877), one DRM (CL1Contig4186) and one DRD1 (CL3348Contig3) proteins.

RdDM is a specialized nuclear branch of RNAi in plants that uses in Arabidopsis DCL3, AGO4, and RDR2. In addition, RdDM requires two plant-specific, RNA polymerase II (Pol II)-related RNA polymerases, called Pol IV and Pol V, as well as a number of accessory factors including putative chromatin remodelers of the defective in RNA-directed DNA methylation 1 (DRD1) subfamily of SNF2 ATPases (Bargsten *et al.* 2013). Pol IV is responsible for generating transcripts that are copied by RDR2 to produce double-stranded RNA precursors, which are then processed into 24-nt siRNAs by DCL3. After being loaded onto AGO4, which interacts with Pol V, the 24-nt siRNAs are thought to base-pair with Pol V-generated scaffold RNAs and guide the DNA methyltansferase domains rearranged methyltransferase-2 (DRM2) to catalyze cytosine methylation at the target DNA region. Methylation occurs at cytosines in all sequence contexts (CG, CHG, and CHH, where H is A, T, or C) and can be maintained at CG and CHG nucleotide groups during subsequent rounds of DNA replication in the absence of the RNA trigger by maintenance methyltransferases methyltransferase-1 (MET1) and chromomethylase-3 (CMT3), respectively (Matzke and Mosher 2014).

**Figure 5:**
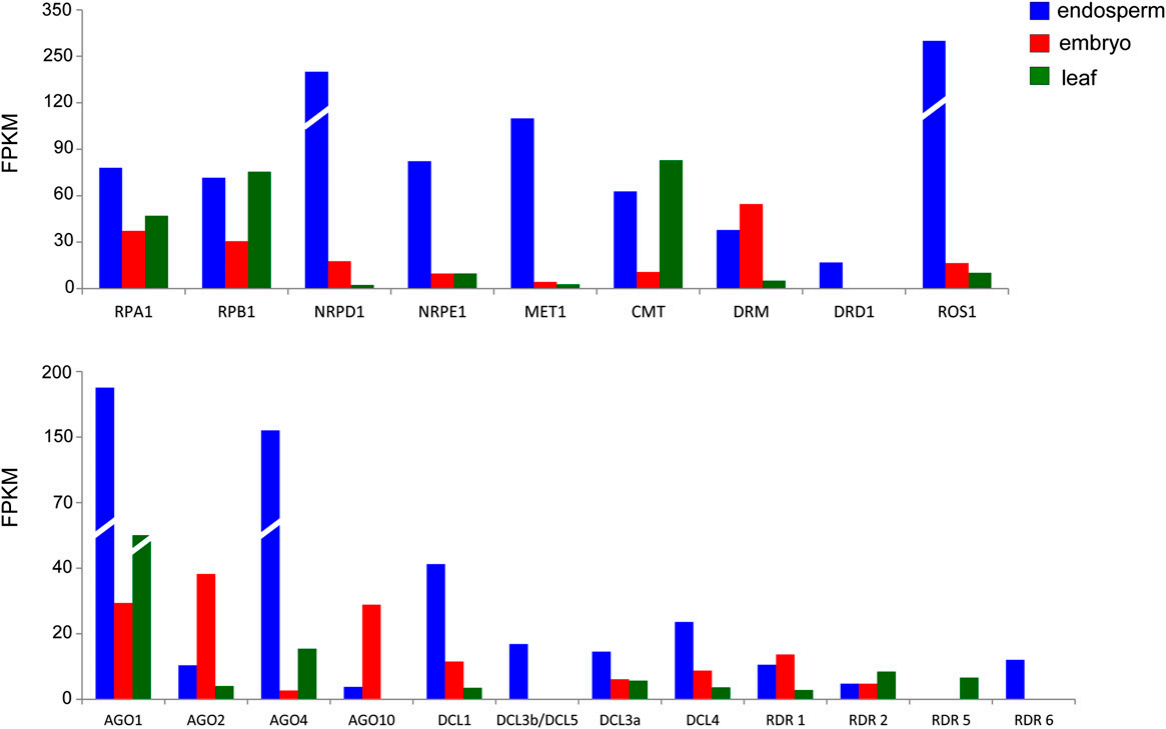
Relative abundance of RdDM-associated gene transcripts found in three tissues of coconut.

We identified partial or nearly full-length transcripts for a number of factors involved in RdDM, including the largest subunits of Pol IV and Pol V (NRPD1 and NRPE1, respectively), as well as AGO4, DRD1, and the three major DNA methyltransferases, MET1, DRM, and CMT3 (Table 3). Notably, the relative abundance of most of these factors is highest in endosperm tissue compared with leaves and embryos (Figure 5). In particular, the relative abundance of the largest subunits of Pol IV and Pol V are higher in endosperm than in embryos or leaves. By contrast, the largest subunits of Pol I (RPA1) and Pol II (RPB1) have similar relative abundances in the three tissues. Although RDR2 is expressed in endosperm, the relative abundance is not overly high compared with the other tissues (Figure 5). Interestingly, a gene encoding a protein similar to the repressor of silencing-1/Demeter (ROS1/DME) family of DNA glycosylase/lyases involved in active DNA demethylation is highly expressed in endosperm. This finding is significant in view of the requirement for DME in establishing parental imprints in Arabidopsis and rice endosperm (Gehring 2013).

**Figure 6:**
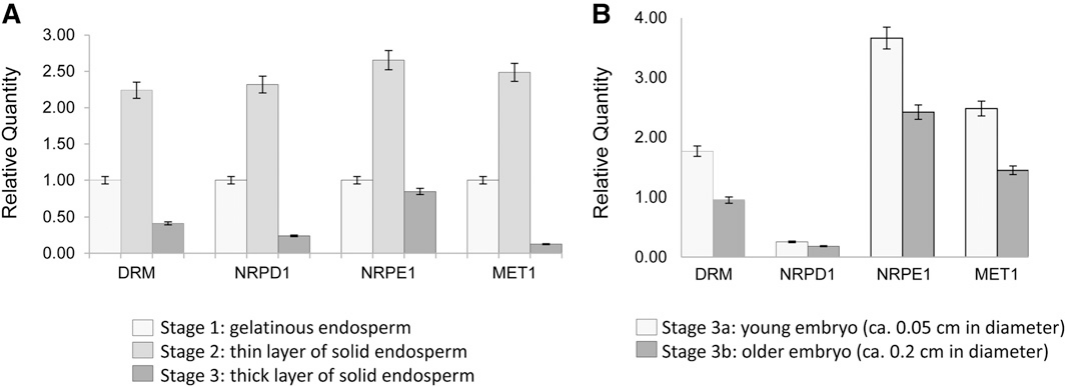
(A) Relative quantity of four RdDM-related genes (DRM, NRPD1, NRPE1, and MET1) in three different developmental stages of endosperm. (B) Relative quantity of the same four genes in two different developmental stages of embryo.

Consistent with the importance of RdDM during seed development, the expression of DRM, NRPD1, NRPE1, and MET1 increases during endosperm maturation but is reduced in fully mature endosperm comprising a thick solid layer (Figure 6A). A decrease in expression of these factors is seen in older embryos as compared with younger embryos (Figure 6B).

Similar to rice (Song *et al.* 2012), transcripts of two DCL3-related enzymes were detected in coconut (Table 3). DCL3a, which is conserved in dicots and monocots (Kapoor *et al.* 2008), was represented by transcripts in all three tissues examined, but the level is highest in endosperm (Figure 5). By contrast, DCL3b/DCL5 (Margis *et al.* 2006; Fei *et al.* 2013), which is monocot-specific and expressed preferentially reproductive tissues and developing seeds (Kapoor *et al.* 2008; Song *et al.* 2012), was detected only in endosperm tissue (Figure 5). In rice, DCL3a is responsible for producing 24-nt siRNAs that induce RdDM, whereas DCL3b/DCL5 produces phased 24-nt siRNAs that are also likely to induce RdDM during reproduction (Song *et al.* 2012; Fei *et al.* 2013). Our results extend the findings of a distinct DCL3b/DCL5 enzyme to developing seeds of a nonmodel monocot plant.

We also identified transcripts of other DCL, AGO, and RDR family members that are involved in small RNA-mediated silencing at the posttranscriptional level. Some of these are also relatively highly expressed in endosperm compared with leaves and embryo. These include DCL1 and AGO1, which act in microRNA pathways important for development, as well as DCL4 and RDR6, which produce endogenous 21-nt siRNAs for viral defense and *trans*-acting siRNAs that influence developmental timing (Willmann *et al.* 2011; Martínez de Alba *et al.* 2013; Poulsen *et al.* 2013). Interestingly, transcripts of AGO2 and AGO10, which act similarly to AGO1 in miRNA pathways but in more specialized contexts (Poulsen *et al.* 2013), are most abundant in embryos. We also detected in one or more of the tested tissues transcripts for RDR proteins that are less well-functionally characterized, including RDR1 and RDR5 (Willmann *et al.* 2011) (Table 3 and Figure 5).

Our results suggest that the RdDM and other small RNA-mediated silencing pathways are active in coconut seeds, particularly maturing endosperm. This finding is consistent with previous studies of other plants indicating that RdDM is late-acting in endosperm development (Belmonte *et al.* 2013; Gehring 2013). Future studies will focus on examining transcription of these factors more thoroughly at different stages of endosperm and embryo development.

## Conclusions

Our study increases transcriptomic resources for coconut and provides a foundation for further functional and molecular studies that will inform efforts to improve coconut through molecular breeding and genetic engineering technologies. Our transcript profiles for leaves, endosperm, and embryos reveal highly expressed genes that can eventually help to identify strong tissue-specific promoters for future use in coconut biotechnology. Our analysis of RdDM factors in coconut expands the range of plants for which sequence information on these proteins is available and broadens our knowledge of epigenetic contributions to seed development in a nonmodel crop plant.

## Supplementary Material

Supporting Information
