# Supplementary material for: De Novo Transcriptome Sequence Assembly from Coconut Leaves and Seeds with a Focus on Factors Involved in RNA-Directed DNA Methylation
Source: G3 (Bethesda). 2014 Sep 4;4(11):2147–57. doi: 10.1534/g3.114.013409 (PMC4232540; doi:10.1534/g3.114.013409)
Supplement: Supporting Information [file supp_4_11_2147__index.html]

De Novo Transcriptome Sequence Assembly from Coconut Leaves and Seeds with a Focus on Factors Involved in RNA-Directed DNA Methylation — Supporting Information 

# *De Novo* Transcriptome Sequence Assembly from Coconut Leaves and Seeds with a Focus on Factors Involved in RNA-Directed DNA Methylation

## Supporting Information for Huang *et al.*, 2014

**Files in this Data Supplement:**

- Supporting Information - Figures S1-S5 and Tables S1-S8 (PDF, 421 KB)
- Figure S1 - Coconut seed development. (PDF, 195 KB)
- Figure S2 - Length distribution of total unigenes found in three coconut tissues. (PDF, 325 KB)
- Figure S3 - Analysis of gene ontology at level two. (PDF, 390 KB)
- Figure S4 - Pie charts demonstrate composition of cellular component of GO annotation at level 8. (PDF, 334 KB)
- Figure S5 - Pie charts demonstrate composition of molecular function of GO annotation at level 8. (PDF, 339 KB)
- Table S1 - List of primers used for sequence validation of factors involved in RNA-directed DNA methylation. (PDF, 113 KB)
- Table S5 - List of complete GO terms for cellular component at level 8. (PDF, 116 KB)
- Table S6 - List of complete GO terms for molecular function at level 8. (PDF, 118 KB)
- Table S7 - List of complete GO terms for biological process at level 8. (PDF, 132 KB)
- Table S8 - List of complete GO terms for biological process at level 8. (PDF, 126 KB)
- Table S2 - Complete gene expression lists (FPKM>1) of the embryo transcriptome. (.xlsx, 2 MB)
- Table S3 - Complete gene expression lists (FPKM>1) of the endosperm transcriptome. (.xlsx, 1 MB)
- Table S4 - Complete gene expression lists (FPKM>1) of the leaf transcriptome. (.xlsx, 1 MB)
