# Supplementary material for: De Novo Transcriptome Sequence Assembly from Coconut Leaves and Seeds with a Focus on Factors Involved in RNA-Directed DNA Methylation
Source: G3 (Bethesda). 2014 Sep 4;4(11):2147–57. doi: 10.1534/g3.114.013409 (PMC4232540; doi:10.1534/g3.114.013409)
Supplement: Supporting Information [file supp_g3.114.013409_013409SI.pdf]

G3·Genes | Genomes | Genetics

De novo transcriptome sequence assembly from coconut leaves and seeds with a focus on factors involved in RNA-directed DNA methylation

Ya-Yi Huang*, Chueh-Pai Lee §, Jason L. Fu*, Bill Chia-Han Chang§, Antonius J.M. Matzke* and Marjori Matzke*

***Institute of Plant and Microbial Biology, Academia Sinica, Taipei, Taiwan**

§Yourgene Bioscience, Taipei, Taiwan

Corresponding authors: Marjori Matzke and Antonius J.M. Matzke, Institute of Plant and Microbial Biology, Academia Sinica, 128, Sec. 2, Academia Road, Nangang, Taipei 11529, Taiwan. Email: marjorimatzke@gate.sinica.edu.tw, antoniusmatzke@gate.sinica.edu.tw

DOI: 10.1534/g3.114.013409

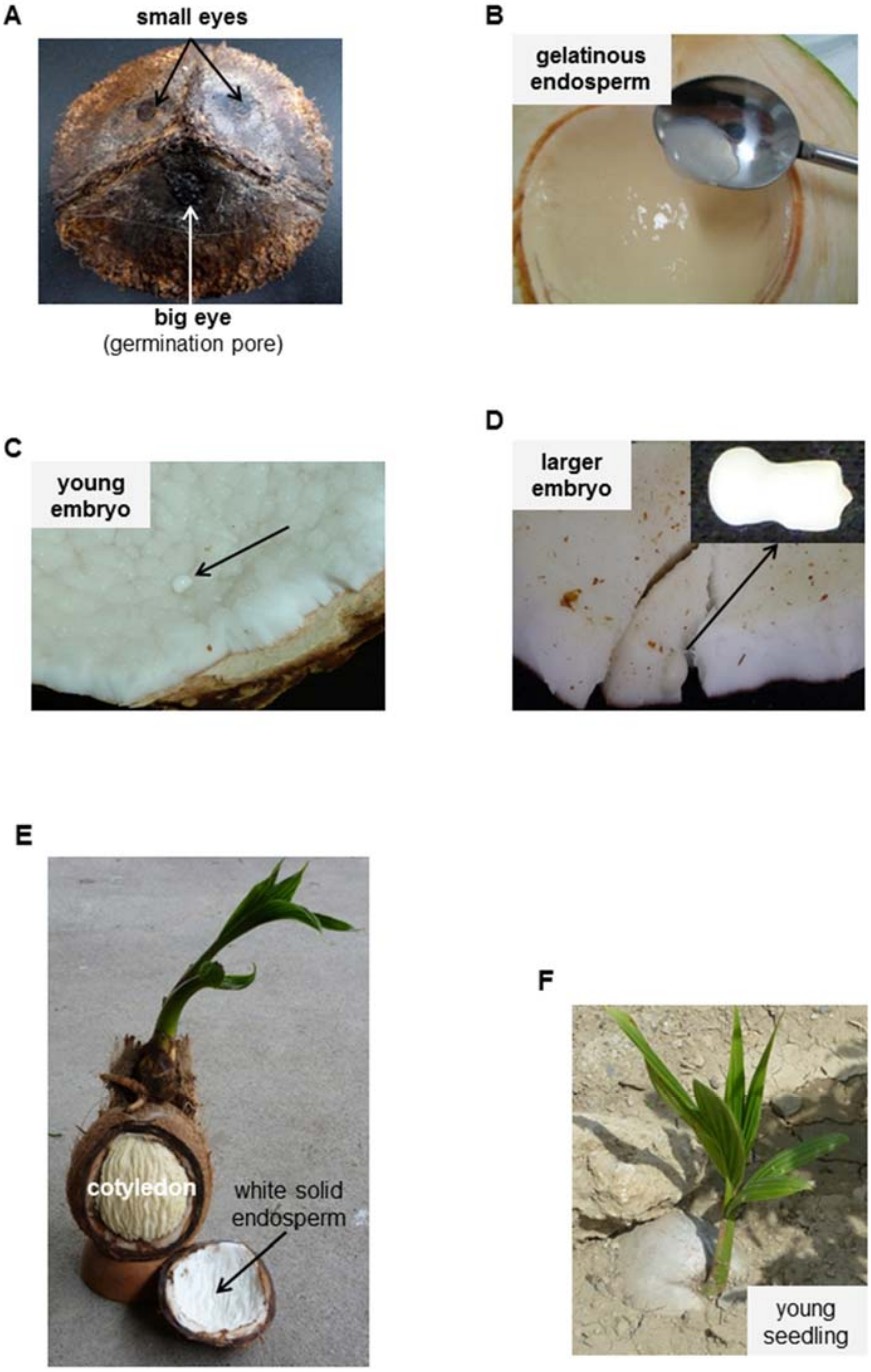

Figure S1 – Coconut seed development. The seeds of flowering plants (angiosperms) contain the products of double fertilization: a diploid embryo containing one maternal (m) and one paternal (p) genome and a triploid endosperm (2m:1p). The embryo grows into the adult plant while the endosperm represents a terminally differentiated tissue that provides nutrients for the developing embryo. In monocots such as coconut, the embryo is very small compared to the endosperm. Coconut has the second largest seed in the world, only after the *Lodoicea maldivica*, another palm species endemic to the Seychelles.

Coconut fruit is a drupe in which exocarp and mesocarp make up the husk while the endocarp forms a

protective hard shell with three uneven germination pores (the eyes). The three eyes (one large, two small) are visible on the outside surface of the shell after the husk is removed (Part A). The mature embryo (around 100 mg) can be found right below the largest eye (soft eye). It is also where the sprout emerges through the husk. Coconut seeds at a very young stage have a cavity within contains abundant aqueous endosperm (coconut water). The liquid endosperm contains free-floating nuclei resulting from a primary nucleus that goes through a process of division without cytokinesis. As the seed development continues, the nuclei move to the periphery and cellularization occurs (Cutter et al., 1952), turning the liquid endosperm first into translucent jelly (Part B) and eventually into up to ten grams of white solid flesh. The solid white flesh only forms a layer on the inner surface of the cavity and never fills it completely.

The embryo, invisible when the endosperm is in liquid form, begins to develop right below the soft eye as the endosperm solidifies. Surrounded by endosperm, the young embryo (ca. 6-month-old) becomes visible at the beginning of endosperm solidification (Part C). As the layer of solid endosperm thickens, the growing embryo is embedded completely in white solid flesh and can only be seen after the flesh is torn apart (Part D). Germination begins when the embryo grows a plumule outwards and a single cotyledon inwards. The plumule eventually pierces through the soft eye and the husk and further develops shoot upwards and root system downwards. The cotyledon absorbs nutrients from both liquid and solid endosperm to support the growth of the plumule. Its tissue becomes spongy and eventually fills up the cavity of the seed (Part E).

Cutter, V.M., Wilson, K.S. and Dube, G.R. 1952. The isolation of living nuclei from the endosperm of *Cocos nucifera*. Science, 115, 58-59.

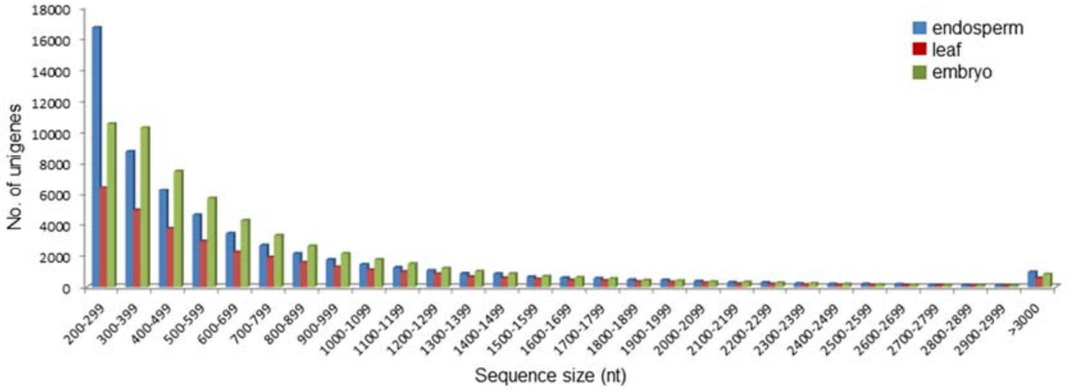

Figure S2 – Length distribution of total unigenes found in three coconut tissues.

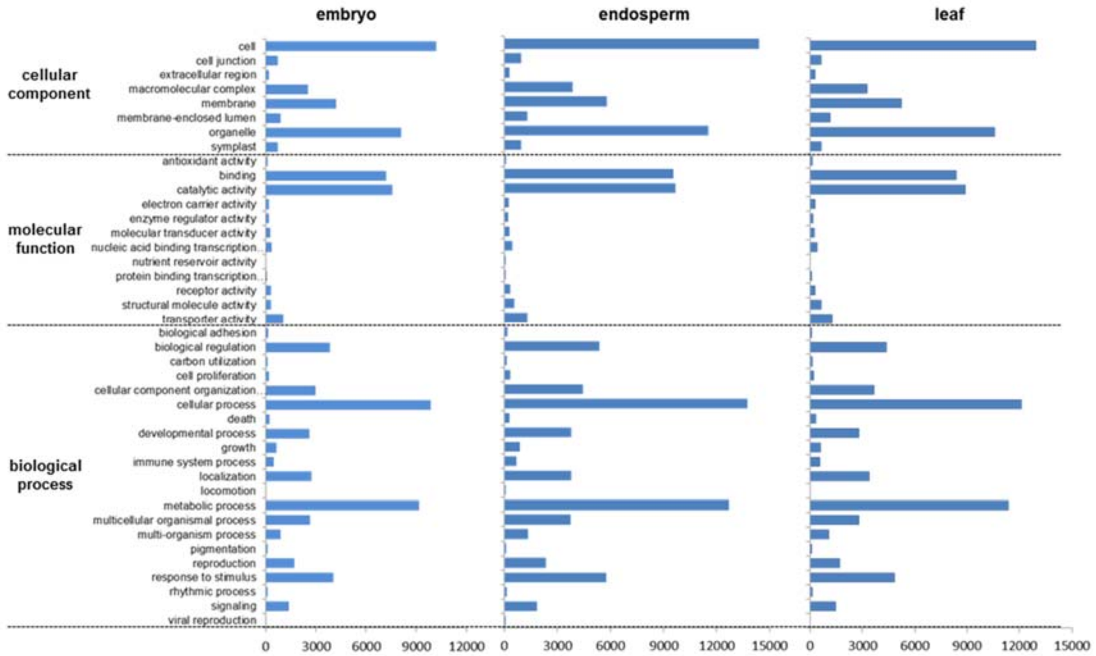

Figure S3. Analysis of gene ontology at level two.

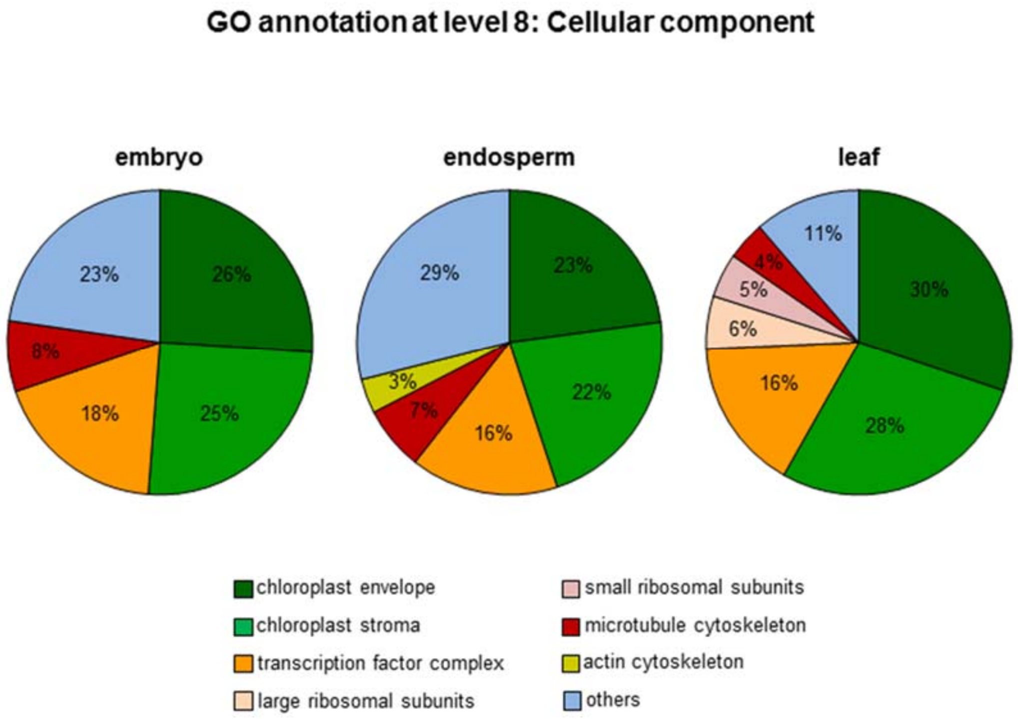

Figure S4 – Pie charts demonstrate composition of cellular component of GO annotation at level 8. Subcategories with unigenes fewer than 100 are merged and placed under the category “others”. For embryo, there are 10 subcategories in the category others while for endosperm and leaf, there are 21 and 6 subcategories, respectively. Leaf transcriptome has higher portions of unigenes involving in the developments of chloroplast and ribosomal subunits, but has relatively fewer unigenes for microtubule cytoskeleton. Endosperm has relatively more proteins involving in the formation of actin cytoskeleton. Moreover, it contains many unique subcategories (denoted as others) that were not annotated for embryo or leaf (Table S5).

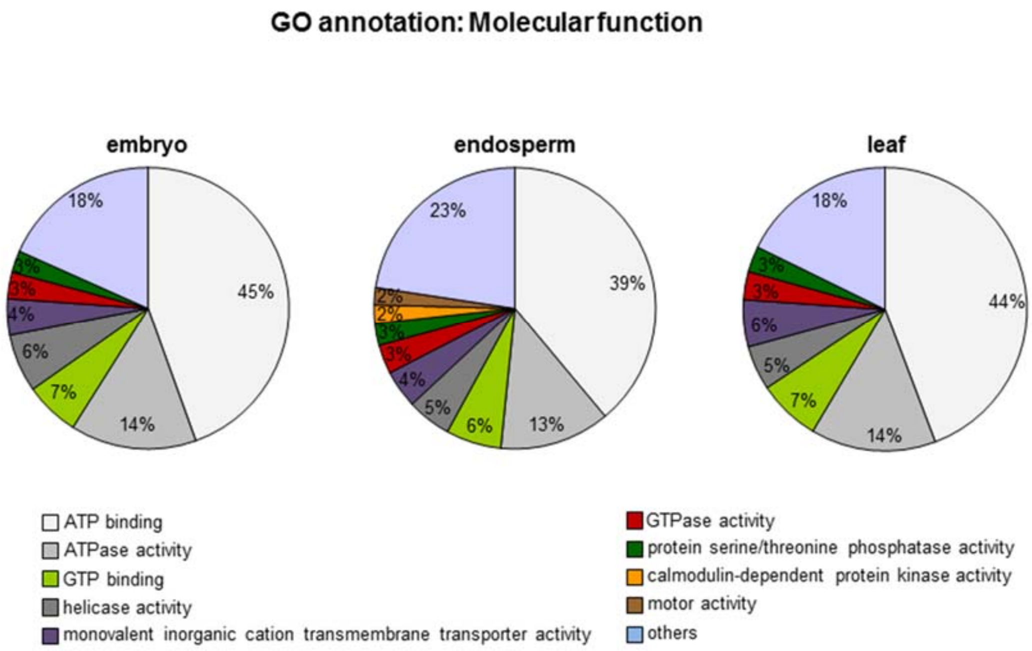

Figure S5 – Pie charts demonstrate composition of molecular function of GO annotation at level 8. Subcategories with unigenes fewer than 100 are merged and placed under the category "others". For embryo, there are 16 subcategories while for endosperm and leaf, there are 33 and 14 subcategories, respectively, in this category. ATP binding and ATPase activity together take up more than half of the GO terms. The highest percentage is found in embryo (59%), followed by leaf (58%), and the least is found in endosperms (52%). Compared to embryo and leaf, endosperm has higher proportion of proteins participating in calmodulin-dependent protein kinase and motor activities. Moreover, as many as 19 subcategories of endosperm were not annotated for embryo or leaf (Table S6).

Table S1 List of primers used for sequence validation of factors involved in RNA-directed DNA methylation

Name	Primer sequence (5' to 3')
RPB1F	TGGATGAGTTGCGTGTGGTTAT
RPBR1	TAGCCAAATGCCGGTAGTTGAC
RPB1R2	GTAGGGCTGTATGAAGGAGAAGTTG
NRPE1F	GAATCACCAGCAAAGCCTGTCT
NRPE1R1	TGCCTCGTGCGATTTCCTT
NRPE1R2	CAGCATTCGTGCCATCACAAG
NRPD2F	CCTCCTGGTTGTATCCTTGGTG
NRPD2R	CGAAAAGACAGCATGCCCAGAC
RDRP6F	CCTGGACACTGCCTGATTCATTC
RDRP6R1	GCTAAAAAGATGATTCCCTCCT
RDRP6R2	GCCCGAATCAAACTCATGCTTG
RDRP6R3	CTTGGTGTCATCAGTAGCGTAATCC
CMTF	CGACGAGACGCCTAGTGTTGATG
CMTR1	CTGATTGGCATTTGCCTTTG
CMTR2	GACACTTGCTTAAACCATCCATAGG
DRDF	GATGTGGTTTGAGTGGAGTGAATATTG
DRDR	CATCTTCTCTCAAAATGGGGCTC
DCL3bF	GAATGACCCTTGACTGCTCTCG
DCL3bR	GAATGACCCTTGACTGCTCTCG
DRMF	TGGTGATAGGCTCGAACACATG
DRMR	GACTTCCGCCAATAACCAGATC
MET1F	ACGAATTCCAAAGCATCCAGGT
METR	GTTCGGTAGGCACCATGGAATC
NRPD1F	TTGCTTATGGCTGCAGATTGC
NRPD1R	TGGAGATGAGATGGATGCTTGA

Tables S2-S4

Available for download as Excel files at

http://www.g3journal.org/lookup/suppl/doi:10.1534/g3.114.013409/-/DC1

Table S2. Complete gene expression lists (FPKM>1) of the embryo transcriptome.

Table S3 Complete gene expression lists (FPKM>1) of the endosperm transcriptome.

Table S4. Complete gene expression lists (FPKM>1) of the leaf transcriptome.

Table S5 List of complete GO terms for cellular component at level 8

GO annotation at level 8	Tissue		
	embryo	endosperm	leaf
actin cytoskeleton	79	123	63
chloroplast envelope	528	768	760
chloroplast stroma	512	741	818
clathrin coated vesicle membrane	-	28	-
clathrin vesicle coat	-	25	-
coated vesicle	71	97	56
condensed chromosome	-	36	-
COPI coated vesicle membrane	-	30	-
COPI-coated vesicle	-	30	-
COPII vesicle coat	-	26	-
DNA-directed RNA polymerase II, holoenzyme	-	41	-
ER to Golgi transport vesicle membrane	-	26	-
glyoxysome	-	21	-
Golgi-associated vesicle	36	49	-
heterotrimeric G-protein complex	47	59	55
histone acetyltransferase complex	57	74	57
large ribosomal subunit	48	88	153
mediator complex	29	50	38
microsome	-	40	37
microtubule cytoskeleton	153	228	111
nuclear body	27	50	-
nuclear chromatin	-	28	-
peroxisomal membrane	-	33	-
small ribosomal subunit	34	94	127
transcription factor complex	376	527	437
transport vesicle	33	47	-

* A dash mark indicates that no unigene is found in that subcategory.

Table S6 List of complete GO terms for molecular function at level 8

GO annotation at level 8	Tissue		
	endosperm	embryo	leaf
3'-5'-exoribonuclease activity	27	-	-
ATP binding	2280	1908	1982
ATPase activity	742	620	639
calcium ion transmembrane transporter activity	50	47	40
calcium-transporting ATPase activity	39	39	-
calmodulin-dependent protein kinase activity	126	66	89
cation:cation antiporter activity	29	30	36
copper ion transmembrane transporter activity	27	-	-
cyclin-dependent protein kinase activity	23	-	-
divalent inorganic cation transmembrane transporter activity	89	70	73
endoribonuclease activity, producing 5'-phosphomonoesters	33	-	-
exoribonuclease activity, producing 5'-phosphomonoesters	40	35	-
glucan endo-1,3-beta-D-glucosidase activity	25	-	-
GTP binding	372	270	320
GTPase activity	197	131	144
helicase activity	298	287	227
histone acetyltransferase activity	70	53	56
histone-lysine N-methyltransferase activity	36	29	
monovalent inorganic cation transmembrane transporter activity	255	179	241
motor activity	115	70	40
non-membrane spanning protein tyrosine kinase activity	78	76	74
organic phosphonate transmembrane transporter activity	27	-	-
organic phosphonate transmembrane-transporting ATPase activity	27	-	-
organophosphate ester transmembrane transporter activity	27	-	-
phosphate ion transmembrane transporter activity	30	-	-
potassium channel activity	39	-	37
potassium ion transmembrane transporter activity	78	68	71
protein serine/threonine phosphatase activity	147	107	131
protein tyrosine phosphatase activity	31	-	-
protein tyrosine/serine/threonine phosphatase activity	24	-	-
proton-transporting ATPase activity, rotational mechanism	61	32	49
Ran GTPase binding	28	-	-
RNA polymerase II carboxy-terminal domain kinase activity	32	-	-
sodium ion transmembrane transporter activity	25	-	-
solute:hydrogen antiporter activity	24	28	37

solute:hydrogen symporter activity	54	48	65
sugar:hydrogen symporter activity	54	47	64
transition metal ion transmembrane transporter activity	71	48	64
trehalose-phosphatase activity	31	-	-
ubiquitin-specific protease activity	27	-	-
voltage-gated anion channel activity	30	-	-
voltage-gated cation channel activity	38	-	-

* A dash mark indicates that no unigene is found in that subcategory.

Table S7 List of complete GO terms for biological process at level 8

GO annotation at level 8	tissue		
	embryo	endosperm	leaf
protein targeting	611	919	784
ribonucleotide metabolic process	555	772	655
chromatin modification	474	719	419
glucose catabolic process	423	704	675
starch metabolic process	402	577	481
nucleoside triphosphate metabolic process	407	562	466
ribonucleoside triphosphate metabolic process	403	558	465
purine ribonucleoside triphosphate metabolic process	402	554	463
modification-dependent protein catabolic process	315	535	392
negative regulation of cellular macromolecule biosynthetic process	330	529	304
negative regulation of transcription, DNA-dependent	322	511	296
protein modification by small protein conjugation	330	507	364
glucan biosynthetic process	327	453	392
gluconeogenesis	278	442	350
purine nucleoside triphosphate catabolic process	326	437	359
positive regulation of transcription, DNA-dependent	287	411	339
RNA splicing	228	408	288
negative regulation of gene expression, epigenetic	246	390	192
leaf development	274	373	318
ncRNA processing	267	361	411
regulation of translation	227	337	282
purine ribonucleoside metabolic process	239	331	255
protein modification by small protein removal	259	320	238
DNA-dependent DNA replication	176	315	171
trichoblast differentiation	237	313	214
pyruvate metabolic process	223	313	383
trichoblast maturation	235	307	209
root hair cell differentiation	235	307	209
posttranscriptional gene silencing by RNA	228	303	185
guanosine-containing compound metabolic process	202	281	212
proteasomal protein catabolic process	169	274	194
rRNA metabolic process	199	274	346
NADP metabolic process	179	272	344
rRNA processing	198	272	343
histone H3-K9 methylation	130	263	122

tRNA metabolic process	211	258	260
purine ribonucleoside catabolic process	186	254	186
GTP catabolic process	181	251	185
guanosine-containing compound catabolic process	181	251	185
proteasome assembly	119	245	179
mRNA processing	147	245	179
thylakoid membrane organization	174	240	304
isopentenyl diphosphate biosynthetic process, mevalonate-independent pathway	175	235	305
production of small RNA involved in gene silencing by RNA	187	230	153
dsRNA fragmentation	187	230	153
cell tip growth	161	219	159
chorismate metabolic process	137	219	161
nuclear import	171	215	169
protein import into nucleus	171	215	168
starch biosynthetic process	157	212	257
protein polymerization	182	206	115
RNA splicing, via endonucleolytic cleavage and ligation	114	202	142
methylation-dependent chromatin silencing	124	199	85
divalent metal ion transport	169	197	192
regulation of DNA replication	86	190	79
regulation of translational initiation	129	190	142
ATP catabolic process	145	186	174
pyrimidine ribonucleotide biosynthetic process	124	185	156
amino acid transport	113	185	154
establishment of protein localization to vacuole	113	185	140
cellulose biosynthetic process	120	184	109
mRNA catabolic process	138	177	102
protein N-linked glycosylation	158	176	104
reciprocal DNA recombination	146	175	109
nuclear-transcribed mRNA catabolic process	135	174	97
chromatin silencing by small RNA	95	171	77
regulation of actin filament polymerization	158	166	89
xylan metabolic process	145	164	106
establishment of protein localization to peroxisome	91	161	152
protein import into peroxisome matrix	89	157	148
positive regulation of actin filament polymerization	150	156	82
RNA splicing, via transesterification reactions	88	155	118
purine ribonucleotide biosynthetic process	102	154	133

chromatin assembly or disassembly	71	154	102
virus induced gene silencing	137	150	90
protein autophosphorylation	92	148	118
regulation of phosphorylation	123	144	116
double-strand break repair	109	143	72
xylan biosynthetic process	130	142	93
glucuronoxylan metabolic process	130	142	93
nuclear mRNA splicing, via spliceosome	76	141	101
regulation of protein phosphorylation	123	140	115
protein myristoylation	93	139	110
N-terminal protein lipidation	93	139	110
regulation of protein kinase activity	123	135	113
photosystem II assembly	93	134	189
DNA packaging	47	130	86
ribonucleoside triphosphate biosynthetic process	77	128	105
nuclear export	101	126	99
lactate metabolic process	78	125	116
purine nucleoside triphosphate biosynthetic process	76	124	103
establishment of protein localization in mitochondrion	75	117	106
glucosinolate biosynthetic process	92	117	128
tricarboxylic acid cycle	69	117	120
transition metal ion transport	92	116	118
lateral root development	77	114	84
mRNA export from nucleus	89	110	86
response to fructose stimulus	77	107	109
mitochondrial ATP synthesis coupled electron transport	56	105	85
phosphatidylinositol biosynthetic process	62	102	80
carotenoid metabolic process	104	100	156
tetraterpenoid metabolic process	104	100	156
recombinational repair	68	98	46
double-strand break repair via homologous recombination	68	98	46
tetraterpenoid biosynthetic process	98	97	152
regulation of purine nucleotide metabolic process	81	95	65
regulation of purine nucleotide catabolic process	81	95	65
regulation of dephosphorylation	58	93	124
sodium ion transport	63	92	93
jasmonic acid metabolic process	57	91	88
protein acetylation	65	90	66

positive regulation of protein modification process	92	89	81
regulation of G2/M transition of mitotic cell cycle	26	89	-
potassium ion transport	79	89	75
peptidyl-lysine modification	67	88	71
regulation of protein dephosphorylation	57	88	119
embryonic meristem initiation	75	88	51
nucleosome assembly	26	88	62
positive regulation of protein kinase activity	86	86	78
positive regulation of phosphate metabolic process	86	86	78
positive regulation of kinase activity	86	86	78
tRNA processing	71	86	70
jasmonic acid biosynthetic process	49	83	73
regulation of translational elongation	60	83	97
establishment of protein localization to chloroplast	66	82	90
histone phosphorylation	39	79	-
cotyledon development	69	79	55
sphingoid metabolic process	50	78	53
activation of MAPKK activity	76	77	69
mitotic recombination	53	75	49
histone acetylation	57	75	58
peptidyl-proline modification	54	75	58
nitrate transport	42	68	78
telomere maintenance in response to DNA damage	51	67	43
ATP hydrolysis coupled proton transport	42	66	57
ATP synthesis coupled proton transport	33	66	51
chlorinated hydrocarbon metabolic process	51	65	62
nucleoside monophosphate metabolic process	48	62	54
reductive tricarboxylic acid cycle	34	60	60
response to glucose stimulus	49	60	59
phosphatidylglycerol biosynthetic process	40	59	88
regulation of Ras protein signal transduction	57	59	46
ribonucleoside monophosphate metabolic process	46	57	50
ribonucleoside monophosphate biosynthetic process	45	56	50
spindle assembly	-	55	-
somatic cell DNA recombination	42	53	-
diterpenoid metabolic process	36	53	47
regulation of Ras GTPase activity	43	49	41
postreplication repair	-	49	38

metal ion homeostasis	49	49	62
regulation of seed germination	39	48	-
glycosaminoglycan biosynthetic process	42	47	47
RNA 3'-end processing	36	47	37
beta-glucan biosynthetic process	33	44	-
(1->3)-beta-D-glucan metabolic process	33	44	-
beta-glucan metabolic process	33	44	-
negative regulation of protein modification process	27	42	-
phosphatidylinositol phosphorylation	41	42	-
lithium ion transport	31	41	38
sphingoid biosynthetic process	-	40	-
ammonium transport	-	39	37
phosphate ion transport	-	38	38
ceramide metabolic process	28	38	-
pyrimidine ribonucleoside metabolic process	27	37	-
terpene catabolic process	-	37	-
trichome branching	-	37	-
monoterpene catabolic process	-	36	-
chloroplast fission	-	36	-
monoterpene metabolic process	-	36	-
limonene metabolic process	-	36	-
positive regulation of flavonoid biosynthetic process	-	35	37
tRNA modification	-	34	-
nucleoside bisphosphate metabolic process	-	34	-
sesquiterpenoid metabolic process	27	34	36
abscisic acid metabolic process	26	33	-
apocarotenoid metabolic process	26	33	-
regulation of ion transmembrane transporter activity	-	33	-
sesquiterpenoid biosynthetic process	-	32	-
nucleotide-excision repair	-	32	-
apocarotenoid biosynthetic process	-	32	-
divalent inorganic cation homeostasis	27	32	38
regulation of transcription from RNA polymerase II promoter	28	32	-
gibberellin metabolic process	-	31	-
purine nucleoside monophosphate biosynthetic process	-	31	-
purine ribonucleoside monophosphate metabolic process	-	31	-
negative regulation of phosphate metabolic process	-	31	-
polar nucleus fusion	-	30	-

heterochromatin organization	-	30	-
starch catabolic process	28	29	-
DNA geometric change	-	28	-
positive regulation of translation	-	28	-
pollen exine formation	30	28	-
halogenated hydrocarbon catabolic process	-	28	-
diterpenoid biosynthetic process	-	28	-
malate metabolic process	-	27	-
organic phosphonate transport	-	27	-
organophosphate ester transport	-	27	-
DNA duplex unwinding	-	26	-
RNA polyadenylation	-	26	-
negative regulation of kinase activity	-	26	-
negative regulation of protein kinase activity	-	26	-
chondroitin sulfate proteoglycan biosynthetic process	-	26	-
chloroplast RNA processing	-	25	-
RNA-dependent DNA replication	-	25	-
mRNA 3'-end processing	-	25	-
protein import into nucleus, docking	-	25	-
triterpenoid biosynthetic process	-	25	-
pyrimidine nucleoside monophosphate biosynthetic process	-	25	-
triterpenoid metabolic process	-	25	-
glycosaminoglycan catabolic process	-	25	-
iron ion homeostasis	-	24	-
base-excision repair	-	24	-
intracellular protein transmembrane transport	-	24	-
peptidyl-tyrosine dephosphorylation	-	24	-
basipetal auxin transport	-	24	-
positive regulation of GTPase activity	-	24	-
protein deacetylation	-	23	-
translational termination	-	23	-
pentacyclic triterpenoid metabolic process	-	22	-
pentacyclic triterpenoid biosynthetic process	-	22	-
pyrimidine ribonucleoside monophosphate metabolic process	-	22	-
purine ribonucleoside biosynthetic process	-	22	-
monovalent inorganic cation homeostasis	-	21	-
chloride transport	-	21	-
mismatch repair	32	21	-

histone deacetylation	-	21	-

* A dash mark indicates that no unigene is found in that subcategory.

Table S8 List of complete GO terms for biological process at level 8

Pathway	embryo	endosperm	leaf
Alanine, aspartate and glutamate metabolism	70	92	93
alpha-Linolenic acid metabolism	43	55	73
Amino sugar and nucleotide sugar metabolism	116	193	158
Aminoacyl-tRNA biosynthesis	76	102	102
Aminobenzoate degradation	53	72	82
Anthocyanin biosynthesis	5	2	9
Arachidonic acid metabolism	33	41	50
Arginine and proline metabolism	113	133	122
Ascorbate and aldarate metabolism	48	66	57
Atrazine degradation	5	4	4
Benzoate degradation	11	30	23
beta-Alanine metabolism	51	68	60
beta-Lactam resistance	1	3	1
Betalain biosynthesis	2	2	2
Biosynthesis of ansamycins	5	9	5
Biosynthesis of siderophore group nonribosomal peptides	0	3	3
Biosynthesis of terpenoids and steroids	1	0	1
Biosynthesis of unsaturated fatty acids	35	59	48
Biosynthesis of vancomycin group antibiotics	3	7	6
Biotin metabolism	18	28	28
Butanoate metabolism	36	53	54
Butirosin and neomycin biosynthesis	5	12	5
C5-Branched dibasic acid metabolism	13	19	20
Caffeine metabolism	9	12	27
Caprolactam degradation	13	22	15
Carbon fixation in photosynthetic organisms	93	140	142
Carbon fixation pathways in prokaryotes	65	104	102
Carotenoid biosynthesis	12	11	13
Chloroalkane and chloroalkene degradation	33	29	23
Chlorocyclohexane and chlorobenzene degradation	6	9	8
Citrate cycle (TCA cycle)	71	105	116
Cutin, suberine and wax biosynthesis	6	4	8
Cyanoamino acid metabolism	27	37	52
Cysteine and methionine metabolism	97	112	112
D-Alanine metabolism	6	4	4
D-Arginine and D-ornithine metabolism	3	2	3

D-Glutamine and D-glutamate metabolism	5	8	4
Diterpenoid biosynthesis	9	5	3
Drug metabolism - cytochrome P450	39	36	46
Drug metabolism - other enzymes	72	92	91
Ether lipid metabolism	34	42	36
Ethylbenzene degradation	3	6	5
Fatty acid biosynthesis	37	68	53
Fatty acid elongation	17	35	25
Fatty acid metabolism	70	94	93
Flavone and flavonol biosynthesis	5	5	13
Flavonoid biosynthesis	31	32	71
Fluorobenzoate degradation	4	8	7
Folate biosynthesis	12	14	14
Fructose and mannose metabolism	65	103	99
Galactose metabolism	89	108	111
Geraniol degradation	18	30	23
Glucosinolate biosynthesis	4	3	5
Glutathione metabolism	92	95	114
Glycerolipid metabolism	130	132	130
Glycerophospholipid metabolism	100	136	110
Glycine, serine and threonine metabolism	90	124	126
Glycolysis Gluconeogenesis	155	245	205
Glycosaminoglycan biosynthesis - chondroitin sulfate dermatan sulfate	22	26	22
Glycosaminoglycan biosynthesis - heparan sulfate heparin	33	39	32
Glycosaminoglycan biosynthesis - keratan sulfate	2	1	0
Glycosaminoglycan degradation	23	25	19
Glycosphingolipid biosynthesis - ganglio series	22	24	17
Glycosphingolipid biosynthesis - globo series	17	18	14
Glycosphingolipid biosynthesis - lacto and neolacto series	4	7	2
Glycosylphosphatidylinositol(GPI)-anchor biosynthesis	3	2	2
Glyoxylate and dicarboxylate metabolism	89	129	154
Histidine metabolism	28	31	27
Indole alkaloid biosynthesis	8	10	15
Inositol phosphate metabolism	86	97	71
Insect hormone biosynthesis	0	0	1
Isoflavonoid biosynthesis	0	2	5
Isoquinoline alkaloid biosynthesis	24	38	31
Limonene and pinene degradation	24	36	26

Linoleic acid metabolism	19	24	54
Lipoic acid metabolism	8	12	9
Lipopolysaccharide biosynthesis	5	11	9
Lysine biosynthesis	21	24	25
Lysine degradation	58	88	61
Metabolism of xenobiotics by cytochrome P450	37	38	49
Methane metabolism	101	240	183
Monoterpenoid biosynthesis	1	5	6
mTOR signaling pathway	17	18	14
Mucin type O-Glycan biosynthesis	2	1	0
Naphthalene degradation	13	7	7
N-Glycan biosynthesis	29	35	27
Nicotinate and nicotinamide metabolism	24	17	30
Nitrogen metabolism	112	190	183
Novobiocin biosynthesis	19	25	17
One carbon pool by folate	32	36	42
Other glycan degradation	52	45	40
Other types of O-glycan biosynthesis	7	7	10
Oxidative phosphorylation	94	163	149
Pantothenate and CoA biosynthesis	35	39	41
Penicillin and cephalosporin biosynthesis	2	4	3
Pentose and glucuronate interconversions	64	101	62
Pentose phosphate pathway	74	114	130
Peptidoglycan biosynthesis	10	31	5
Phenylalanine metabolism	100	139	114
Phenylalanine, tyrosine and tryptophan biosynthesis	69	84	77
Phenylpropanoid biosynthesis	53	77	84
Phosphatidylinositol signaling system	109	107	74
Phosphonate and phosphinate metabolism	2	9	5
Photosynthesis	3	5	3
Polyketide sugar unit biosynthesis	7	11	10
Porphyrin and chlorophyll metabolism	65	73	87
Primary bile acid biosynthesis	5	13	5
Propanoate metabolism	55	83	75
Purine metabolism	351	415	412
Pyrimidine metabolism	148	197	172
Pyruvate metabolism	107	162	170
Retinol metabolism	22	22	37

Riboflavin metabolism	31	44	45
Selenocompound metabolism	36	38	37
Sesquiterpenoid and triterpenoid biosynthesis	1	0	1
Sphingolipid metabolism	72	70	63
Starch and sucrose metabolism	274	409	272
Steroid biosynthesis	19	37	34
Steroid degradation	7	6	8
Steroid hormone biosynthesis	11	32	40
Stilbenoid, diarylheptanoid and gingerol biosynthesis	4	6	10
Streptomycin biosynthesis	18	47	38
Styrene degradation	17	18	19
Sulfur metabolism	50	64	68
Synthesis and degradation of ketone bodies	8	10	14
T cell receptor signaling pathway	120	137	118
Taurine and hypotaurine metabolism	7	9	8
Terpenoid backbone biosynthesis	33	52	57
Tetracycline biosynthesis	9	14	12
Thiamine metabolism	79	78	81
Toluene degradation	11	19	17
Tropane, piperidine and pyridine alkaloid biosynthesis	28	42	29
Tryptophan metabolism	71	88	87
Tyrosine metabolism	53	68	52
Ubiquinone and other terpenoid-quinone biosynthesis	23	31	31
Valine, leucine and isoleucine biosynthesis	19	25	22
Valine, leucine and isoleucine degradation	66	87	87
Various types of N-glycan biosynthesis	22	26	20
Vitamin B6 metabolism	8	21	17
Xylene degradation	3	1	2
Zeatin biosynthesis	4	6	8
**Total**	**5686**	**7707**	**7231**
