# Supplementary material for: De Novo Transcriptome Sequence Assembly from Coconut Leaves and Seeds with a Focus on Factors Involved in RNA-Directed DNA Methylation
Source: G3 (Bethesda). 2014 Sep 4;4(11):2147–57. doi: 10.1534/g3.114.013409 (PMC4232540; doi:10.1534/g3.114.013409)
Supplement: Supporting Information [file supp_g3.114.013409_FigureS1.pdf]

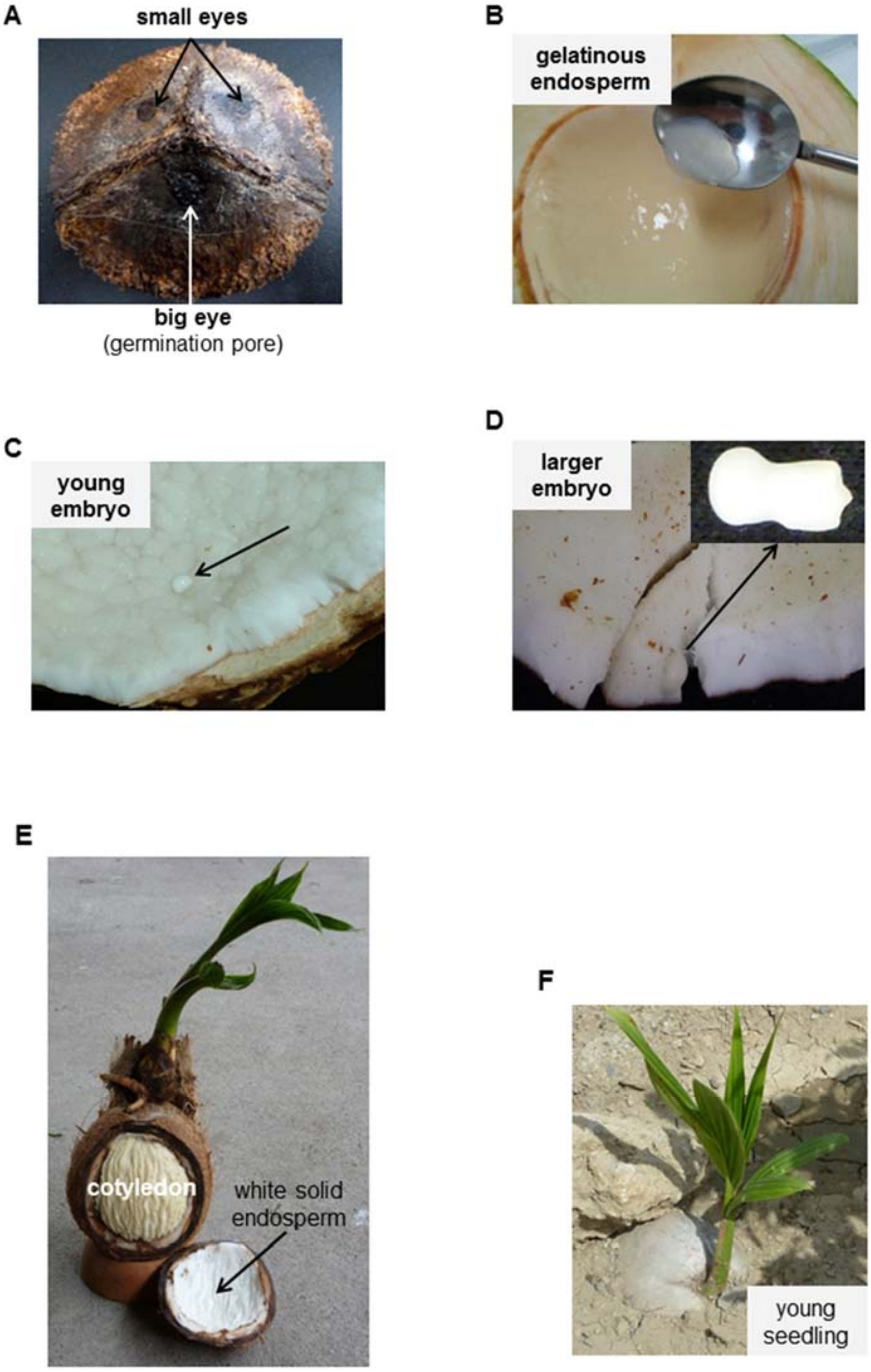

Figure S1 – Coconut seed development. The seeds of flowering plants (angiosperms) contain the products of double fertilization: a diploid embryo containing one maternal (m) and one paternal (p) genome and a triploid endosperm (2m:1p). The embryo grows into the adult plant while the endosperm represents a terminally differentiated tissue that provides nutrients for the developing embryo. In monocots such as coconut, the embryo is very small compared to the endosperm. Coconut has the second largest seed in the world, only after the *Lodoicea maldivica*, another palm species endemic to the Seychelles.

Coconut fruit is a drupe in which exocarp and mesocarp make up the husk while the endocarp forms a

protective hard shell with three uneven germination pores (the eyes). The three eyes (one large, two small) are visible on the outside surface of the shell after the husk is removed (Part A). The mature embryo (around 100 mg) can be found right below the largest eye (soft eye). It is also where the sprout emerges through the husk. Coconut seeds at a very young stage have a cavity within contains abundant aqueous endosperm (coconut water). The liquid endosperm contains free-floating nuclei resulting from a primary nucleus that goes through a process of division without cytokinesis. As the seed development continues, the nuclei move to the periphery and cellularization occurs (Cutter et al., 1952), turning the liquid endosperm first into translucent jelly (Part B) and eventually into up to ten grams of white solid flesh. The solid white flesh only forms a layer on the inner surface of the cavity and never fills it completely.

The embryo, invisible when the endosperm is in liquid form, begins to develop right below the soft eye as the endosperm solidifies. Surrounded by endosperm, the young embryo (ca. 6-month-old) becomes visible at the beginning of endosperm solidification (Part C). As the layer of solid endosperm thickens, the growing embryo is embedded completely in white solid flesh and can only be seen after the flesh is torn apart (Part D). Germination begins when the embryo grows a plumule outwards and a single cotyledon inwards. The plumule eventually pierces through the soft eye and the husk and further develops shoot upwards and root system downwards. The cotyledon absorbs nutrients from both liquid and solid endosperm to support the growth of the plumule. Its tissue becomes spongy and eventually fills up the cavity of the seed (Part E).

Cutter, V.M., Wilson, K.S. and Dube, G.R. 1952. The isolation of living nuclei from the endosperm of *Cocos nucifera*. Science, 115, 58-59.
