# Supplementary figures and images for: De Novo Transcriptome Sequence Assembly from Coconut Leaves and Seeds with a Focus on Factors Involved in RNA-Directed DNA Methylation
Source: G3 (Bethesda). 2014 Sep 4;4(11):2147–57. doi: 10.1534/g3.114.013409 (PMC4232540; doi:10.1534/g3.114.013409)

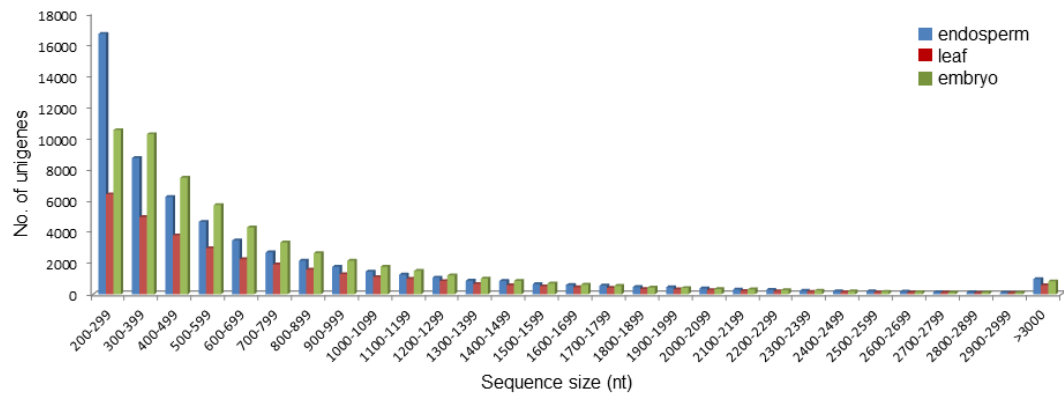

**Figure S2** – Length distribution of total unigenes found in three coconut tissues.

Supplement: Supporting Information [file supp_g3.114.013409_FigureS2.pdf]

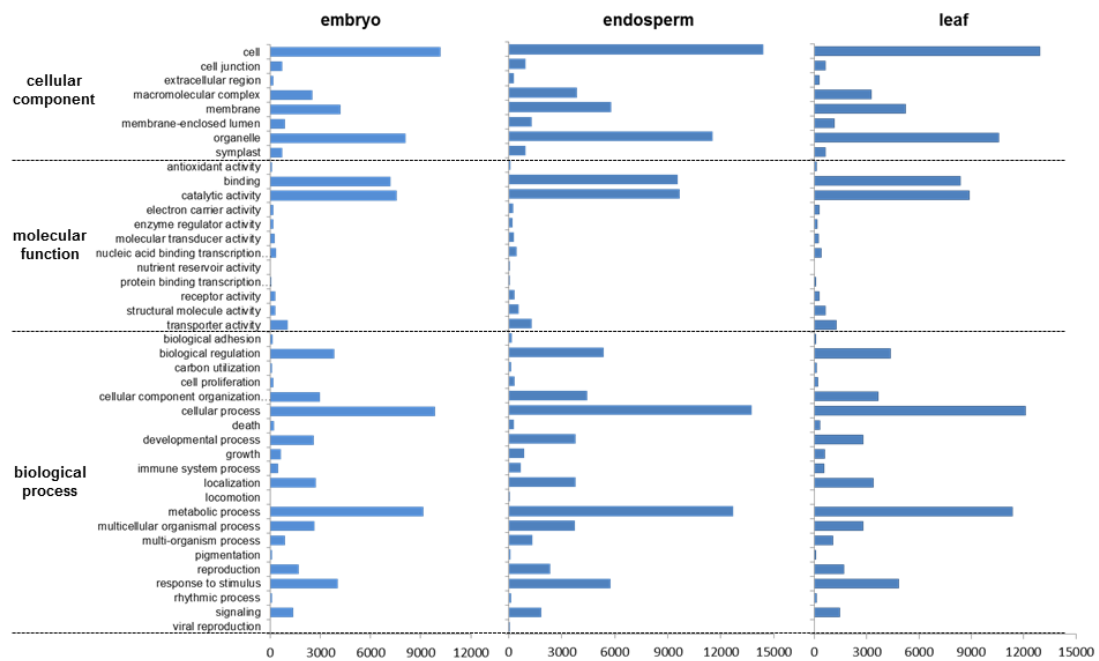

**Figure S3.** Analysis of gene ontology at level two.

Supplement: Supporting Information [file supp_g3.114.013409_FigureS3.pdf]
