# Supplementary material for: De Novo Transcriptome Sequence Assembly from Coconut Leaves and Seeds with a Focus on Factors Involved in RNA-Directed DNA Methylation
Source: G3 (Bethesda). 2014 Sep 4;4(11):2147–57. doi: 10.1534/g3.114.013409 (PMC4232540; doi:10.1534/g3.114.013409)
Supplement: Supporting Information [file supp_g3.114.013409_FigureS4.pdf]

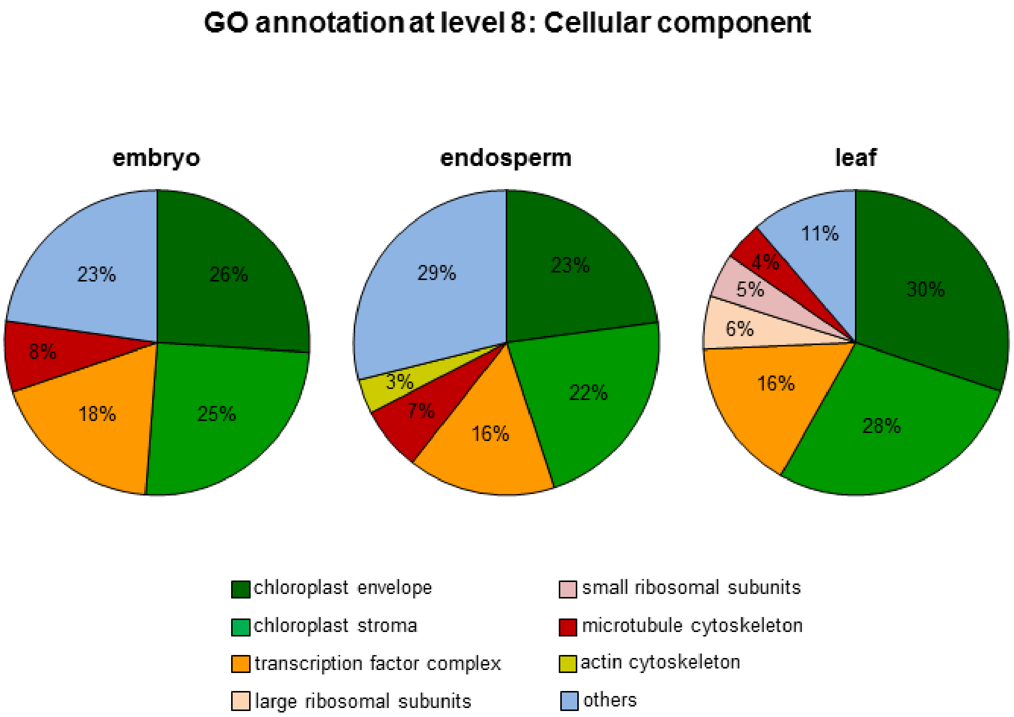

Figure S4 – Pie charts demonstrate composition of cellular component of GO annotation at level 8. Subcategories with unigenes fewer than 100 are merged and placed under the category "others". For embryo, there are 10 subcategories in the category others while for endosperm and leaf, there are 21 and 6 subcategories, respectively. Leaf transcriptome has higher portions of unigenes involving in the developments of chloroplast and ribosomal subunits, but has relatively fewer unigenes for microtubule cytoskeleton. Endosperm has relatively more proteins involving in the formation of actin cytoskeleton. Moreover, it contains many unique subcategories (denoted as others) that were not annotated for embryo or leaf (Table S5).
