# Supplementary material for: De Novo Transcriptome Sequence Assembly from Coconut Leaves and Seeds with a Focus on Factors Involved in RNA-Directed DNA Methylation
Source: G3 (Bethesda). 2014 Sep 4;4(11):2147–57. doi: 10.1534/g3.114.013409 (PMC4232540; doi:10.1534/g3.114.013409)
Supplement: Supporting Information [file supp_g3.114.013409_FigureS5.pdf]

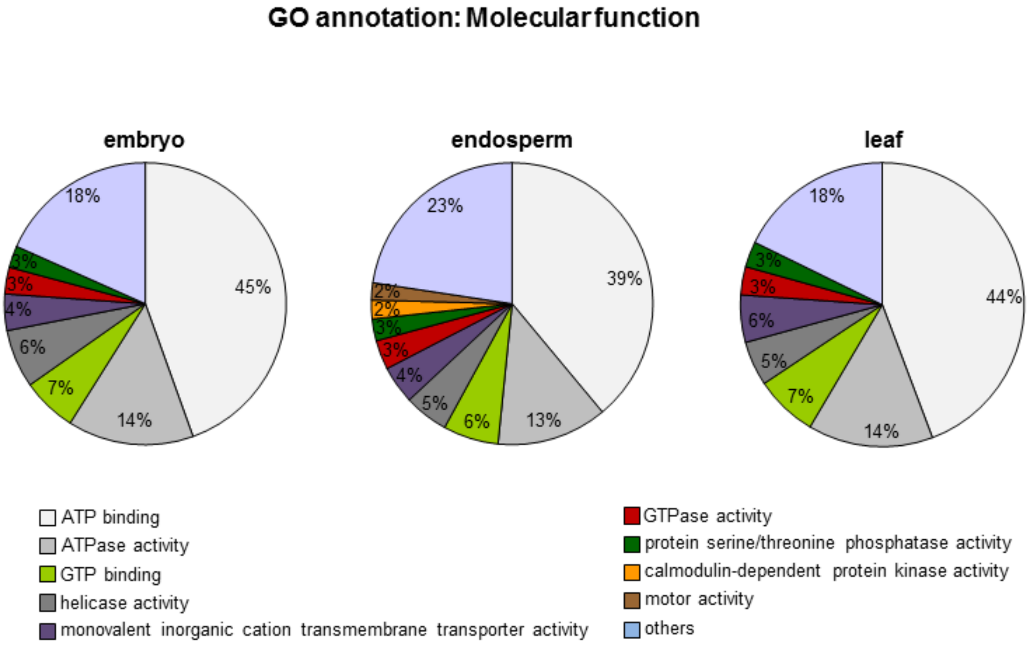

Figure S5 – Pie charts demonstrate composition of molecular function of GO annotation at level 8. Subcategories with unigenes fewer than 100 are merged and placed under the category "others". For embryo, there are 16 subcategories while for endosperm and leaf, there are 33 and 14 subcategories, respectively, in this category. ATP binding and ATPase activity together take up more than half of the GO terms. The highest percentage is found in embryo (59%), followed by leaf (58%), and the least is found in endosperms (52%). Compared to embryo and leaf, endosperm has higher proportion of proteins participating in calmodulin-dependent protein kinase and motor activities. Moreover, as many as 19 subcategories of endosperm were not annotated for embryo or leaf (Table S6).
