# Supplementary material for: De Novo Transcriptome Sequence Assembly from Coconut Leaves and Seeds with a Focus on Factors Involved in RNA-Directed DNA Methylation
Source: G3 (Bethesda). 2014 Sep 4;4(11):2147–57. doi: 10.1534/g3.114.013409 (PMC4232540; doi:10.1534/g3.114.013409)
Supplement: Supporting Information [file supp_g3.114.013409_TableS1.pdf]

Table S1 List of primers used for sequence validation of factors involved in RNA-directed DNA methylation

Name	Primer sequence (5' to 3')
RPB1F	TGGATGAGTTGCGTGTGGTTAT
RPBR1	TAGCCAAATGCCGGTAGTTGAC
RPB1R2	GTAGGGCTGTATGAAGGAGAAGTTG
NRPE1F	GAATCACCAGCAAAGCCTGTCT
NRPE1R1	TGCCTCGTGCGATTTCCTT
NRPE1R2	CAGCATTCGTGCCATCACAAG
NRPD2F	CCTCCTGGTTGTATCCTTGGTG
NRPD2R	CGAAAAGACAGCATGCCCAGAC
RDRP6F	CCTGGACACTGCCTGATTCATTC
RDRP6R1	GCTAAAAAGATGATTCCCTCCT
RDRP6R2	GCCCGAATCAAACTCATGCTTG
RDRP6R3	CTTGGTGTCATCAGTAGCGTAATCC
CMTF	CGACGAGACGCCTAGTGTTGATG
CMTR1	CTGATTGGCATTTGCCTTTG
CMTR2	GACACTTGCTTAAACCATCCATAGG
DRDF	GATGTGGTTTGAGTGGAGTGAATATTG
DRDR	CATCTTCTCTCAAAATGGGGCTC
DCL3bF	GAATGACCCTTGACTGCTCTCG
DCL3bR	GAATGACCCTTGACTGCTCTCG
DRMF	TGGTGATAGGCTCGAACACATG
DRMR	GACTTCCGCCAATAACCAGATC
MET1F	ACGAATTCCAAAGCATCCAGGT
METR	GTTCGGTAGGCACCATGGAATC
NRPD1F	TTGCTTATGGCTGCAGATTGC
NRPD1R	TGGAGATGAGATGGATGCTTGA
