# Supplementary material for: De Novo Transcriptome Sequence Assembly from Coconut Leaves and Seeds with a Focus on Factors Involved in RNA-Directed DNA Methylation
Source: G3 (Bethesda). 2014 Sep 4;4(11):2147–57. doi: 10.1534/g3.114.013409 (PMC4232540; doi:10.1534/g3.114.013409)
Supplement: Supporting Information [file supp_g3.114.013409_TableS5.pdf]

Table S5 List of complete GO terms for cellular component at level 8

GO annotation at level 8	Tissue		
	embryo	endosperm	leaf
actin cytoskeleton	79	123	63
chloroplast envelope	528	768	760
chloroplast stroma	512	741	818
clathrin coated vesicle membrane	-	28	-
clathrin vesicle coat	-	25	-
coated vesicle	71	97	56
condensed chromosome	-	36	-
COPI coated vesicle membrane	-	30	-
COPI-coated vesicle	-	30	-
COPII vesicle coat	-	26	-
DNA-directed RNA polymerase II, holoenzyme	-	41	-
ER to Golgi transport vesicle membrane	-	26	-
glyoxysome	-	21	-
Golgi-associated vesicle	36	49	-
heterotrimeric G-protein complex	47	59	55
histone acetyltransferase complex	57	74	57
large ribosomal subunit	48	88	153
mediator complex	29	50	38
microsome	-	40	37
microtubule cytoskeleton	153	228	111
nuclear body	27	50	-
nuclear chromatin	-	28	-
peroxisomal membrane	-	33	-
small ribosomal subunit	34	94	127
transcription factor complex	376	527	437
transport vesicle	33	47	-

* A dash mark indicates that no unigene is found in that subcategory.
