# Supplementary material for: De Novo Transcriptome Sequence Assembly from Coconut Leaves and Seeds with a Focus on Factors Involved in RNA-Directed DNA Methylation
Source: G3 (Bethesda). 2014 Sep 4;4(11):2147–57. doi: 10.1534/g3.114.013409 (PMC4232540; doi:10.1534/g3.114.013409)
Supplement: Supporting Information [file supp_g3.114.013409_TableS6.pdf]

Table S6 List of complete GO terms for molecular function at level 8

GO annotation at level 8	Tissue		
	endosperm	embryo	leaf
3'-5'-exoribonuclease activity	27	-	-
ATP binding	2280	1908	1982
ATPase activity	742	620	639
calcium ion transmembrane transporter activity	50	47	40
calcium-transporting ATPase activity	39	39	-
calmodulin-dependent protein kinase activity	126	66	89
cation:cation antiporter activity	29	30	36
copper ion transmembrane transporter activity	27	-	-
cyclin-dependent protein kinase activity	23	-	-
divalent inorganic cation transmembrane transporter activity	89	70	73
endoribonuclease activity, producing 5'-phosphomonoesters	33	-	-
exoribonuclease activity, producing 5'-phosphomonoesters	40	35	-
glucan endo-1,3-beta-D-glucosidase activity	25	-	-
GTP binding	372	270	320
GTPase activity	197	131	144
helicase activity	298	287	227
histone acetyltransferase activity	70	53	56
histone-lysine N-methyltransferase activity	36	29	
monovalent inorganic cation transmembrane transporter activity	255	179	241
motor activity	115	70	40
non-membrane spanning protein tyrosine kinase activity	78	76	74
organic phosphonate transmembrane transporter activity	27	-	-
organic phosphonate transmembrane-transporting ATPase activity	27	-	-
organophosphate ester transmembrane transporter activity	27	-	-
phosphate ion transmembrane transporter activity	30	-	-
potassium channel activity	39	-	37
potassium ion transmembrane transporter activity	78	68	71
protein serine/threonine phosphatase activity	147	107	131
protein tyrosine phosphatase activity	31	-	-
protein tyrosine/serine/threonine phosphatase activity	24	-	-
proton-transporting ATPase activity, rotational mechanism	61	32	49
Ran GTPase binding	28	-	-
RNA polymerase II carboxy-terminal domain kinase activity	32	-	-
sodium ion transmembrane transporter activity	25	-	-
solute:hydrogen antiporter activity	24	28	37

solute:hydrogen symporter activity	54	48	65
sugar:hydrogen symporter activity	54	47	64
transition metal ion transmembrane transporter activity	71	48	64
trehalose-phosphatase activity	31	-	-
ubiquitin-specific protease activity	27	-	-
voltage-gated anion channel activity	30	-	-
voltage-gated cation channel activity	38	-	-

* A dash mark indicates that no unigene is found in that subcategory.
