# Supplementary material for: De Novo Transcriptome Sequence Assembly from Coconut Leaves and Seeds with a Focus on Factors Involved in RNA-Directed DNA Methylation
Source: G3 (Bethesda). 2014 Sep 4;4(11):2147–57. doi: 10.1534/g3.114.013409 (PMC4232540; doi:10.1534/g3.114.013409)
Supplement: Supporting Information [file supp_g3.114.013409_TableS7.pdf]

Table S7 List of complete GO terms for biological process at level 8

GO annotation at level 8	tissue		
	embryo	endosperm	leaf
protein targeting	611	919	784
ribonucleotide metabolic process	555	772	655
chromatin modification	474	719	419
glucose catabolic process	423	704	675
starch metabolic process	402	577	481
nucleoside triphosphate metabolic process	407	562	466
ribonucleoside triphosphate metabolic process	403	558	465
purine ribonucleoside triphosphate metabolic process	402	554	463
modification-dependent protein catabolic process	315	535	392
negative regulation of cellular macromolecule biosynthetic process	330	529	304
negative regulation of transcription, DNA-dependent	322	511	296
protein modification by small protein conjugation	330	507	364
glucan biosynthetic process	327	453	392
gluconeogenesis	278	442	350
purine nucleoside triphosphate catabolic process	326	437	359
positive regulation of transcription, DNA-dependent	287	411	339
RNA splicing	228	408	288
negative regulation of gene expression, epigenetic	246	390	192
leaf development	274	373	318
ncRNA processing	267	361	411
regulation of translation	227	337	282
purine ribonucleoside metabolic process	239	331	255
protein modification by small protein removal	259	320	238
DNA-dependent DNA replication	176	315	171
trichoblast differentiation	237	313	214
pyruvate metabolic process	223	313	383
trichoblast maturation	235	307	209
root hair cell differentiation	235	307	209
posttranscriptional gene silencing by RNA	228	303	185
guanosine-containing compound metabolic process	202	281	212
proteasomal protein catabolic process	169	274	194
rRNA metabolic process	199	274	346
NADP metabolic process	179	272	344
rRNA processing	198	272	343
histone H3-K9 methylation	130	263	122

tRNA metabolic process	211	258	260
purine ribonucleoside catabolic process	186	254	186
GTP catabolic process	181	251	185
guanosine-containing compound catabolic process	181	251	185
proteasome assembly	119	245	179
mRNA processing	147	245	179
thylakoid membrane organization	174	240	304
isopentenyl diphosphate biosynthetic process, mevalonate-independent pathway	175	235	305
production of small RNA involved in gene silencing by RNA	187	230	153
dsRNA fragmentation	187	230	153
cell tip growth	161	219	159
chorismate metabolic process	137	219	161
nuclear import	171	215	169
protein import into nucleus	171	215	168
starch biosynthetic process	157	212	257
protein polymerization	182	206	115
RNA splicing, via endonucleolytic cleavage and ligation	114	202	142
methylation-dependent chromatin silencing	124	199	85
divalent metal ion transport	169	197	192
regulation of DNA replication	86	190	79
regulation of translational initiation	129	190	142
ATP catabolic process	145	186	174
pyrimidine ribonucleotide biosynthetic process	124	185	156
amino acid transport	113	185	154
establishment of protein localization to vacuole	113	185	140
cellulose biosynthetic process	120	184	109
mRNA catabolic process	138	177	102
protein N-linked glycosylation	158	176	104
reciprocal DNA recombination	146	175	109
nuclear-transcribed mRNA catabolic process	135	174	97
chromatin silencing by small RNA	95	171	77
regulation of actin filament polymerization	158	166	89
xylan metabolic process	145	164	106
establishment of protein localization to peroxisome	91	161	152
protein import into peroxisome matrix	89	157	148
positive regulation of actin filament polymerization	150	156	82
RNA splicing, via transesterification reactions	88	155	118
purine ribonucleotide biosynthetic process	102	154	133

chromatin assembly or disassembly	71	154	102
virus induced gene silencing	137	150	90
protein autophosphorylation	92	148	118
regulation of phosphorylation	123	144	116
double-strand break repair	109	143	72
xylan biosynthetic process	130	142	93
glucuronoxylan metabolic process	130	142	93
nuclear mRNA splicing, via spliceosome	76	141	101
regulation of protein phosphorylation	123	140	115
protein myristoylation	93	139	110
N-terminal protein lipidation	93	139	110
regulation of protein kinase activity	123	135	113
photosystem II assembly	93	134	189
DNA packaging	47	130	86
ribonucleoside triphosphate biosynthetic process	77	128	105
nuclear export	101	126	99
lactate metabolic process	78	125	116
purine nucleoside triphosphate biosynthetic process	76	124	103
establishment of protein localization in mitochondrion	75	117	106
glucosinolate biosynthetic process	92	117	128
tricarboxylic acid cycle	69	117	120
transition metal ion transport	92	116	118
lateral root development	77	114	84
mRNA export from nucleus	89	110	86
response to fructose stimulus	77	107	109
mitochondrial ATP synthesis coupled electron transport	56	105	85
phosphatidylinositol biosynthetic process	62	102	80
carotenoid metabolic process	104	100	156
tetraterpenoid metabolic process	104	100	156
recombinational repair	68	98	46
double-strand break repair via homologous recombination	68	98	46
tetraterpenoid biosynthetic process	98	97	152
regulation of purine nucleotide metabolic process	81	95	65
regulation of purine nucleotide catabolic process	81	95	65
regulation of dephosphorylation	58	93	124
sodium ion transport	63	92	93
jasmonic acid metabolic process	57	91	88
protein acetylation	65	90	66

positive regulation of protein modification process	92	89	81
regulation of G2/M transition of mitotic cell cycle	26	89	-
potassium ion transport	79	89	75
peptidyl-lysine modification	67	88	71
regulation of protein dephosphorylation	57	88	119
embryonic meristem initiation	75	88	51
nucleosome assembly	26	88	62
positive regulation of protein kinase activity	86	86	78
positive regulation of phosphate metabolic process	86	86	78
positive regulation of kinase activity	86	86	78
tRNA processing	71	86	70
jasmonic acid biosynthetic process	49	83	73
regulation of translational elongation	60	83	97
establishment of protein localization to chloroplast	66	82	90
histone phosphorylation	39	79	-
cotyledon development	69	79	55
sphingoid metabolic process	50	78	53
activation of MAPKK activity	76	77	69
mitotic recombination	53	75	49
histone acetylation	57	75	58
peptidyl-proline modification	54	75	58
nitrate transport	42	68	78
telomere maintenance in response to DNA damage	51	67	43
ATP hydrolysis coupled proton transport	42	66	57
ATP synthesis coupled proton transport	33	66	51
chlorinated hydrocarbon metabolic process	51	65	62
nucleoside monophosphate metabolic process	48	62	54
reductive tricarboxylic acid cycle	34	60	60
response to glucose stimulus	49	60	59
phosphatidylglycerol biosynthetic process	40	59	88
regulation of Ras protein signal transduction	57	59	46
ribonucleoside monophosphate metabolic process	46	57	50
ribonucleoside monophosphate biosynthetic process	45	56	50
spindle assembly	-	55	-
somatic cell DNA recombination	42	53	-
diterpenoid metabolic process	36	53	47
regulation of Ras GTPase activity	43	49	41
postreplication repair	-	49	38

metal ion homeostasis	49	49	62
regulation of seed germination	39	48	-
glycosaminoglycan biosynthetic process	42	47	47
RNA 3'-end processing	36	47	37
beta-glucan biosynthetic process	33	44	-
(1->3)-beta-D-glucan metabolic process	33	44	-
beta-glucan metabolic process	33	44	-
negative regulation of protein modification process	27	42	-
phosphatidylinositol phosphorylation	41	42	-
lithium ion transport	31	41	38
sphingoid biosynthetic process	-	40	-
ammonium transport	-	39	37
phosphate ion transport	-	38	38
ceramide metabolic process	28	38	-
pyrimidine ribonucleoside metabolic process	27	37	-
terpene catabolic process	-	37	-
trichome branching	-	37	-
monoterpene catabolic process	-	36	-
chloroplast fission	-	36	-
monoterpene metabolic process	-	36	-
limonene metabolic process	-	36	-
positive regulation of flavonoid biosynthetic process	-	35	37
tRNA modification	-	34	-
nucleoside bisphosphate metabolic process	-	34	-
sesquiterpenoid metabolic process	27	34	36
abscisic acid metabolic process	26	33	-
apocarotenoid metabolic process	26	33	-
regulation of ion transmembrane transporter activity	-	33	-
sesquiterpenoid biosynthetic process	-	32	-
nucleotide-excision repair	-	32	-
apocarotenoid biosynthetic process	-	32	-
divalent inorganic cation homeostasis	27	32	38
regulation of transcription from RNA polymerase II promoter	28	32	-
gibberellin metabolic process	-	31	-
purine nucleoside monophosphate biosynthetic process	-	31	-
purine ribonucleoside monophosphate metabolic process	-	31	-
negative regulation of phosphate metabolic process	-	31	-
polar nucleus fusion	-	30	-

heterochromatin organization	-	30	-
starch catabolic process	28	29	-
DNA geometric change	-	28	-
positive regulation of translation	-	28	-
pollen exine formation	30	28	-
halogenated hydrocarbon catabolic process	-	28	-
diterpenoid biosynthetic process	-	28	-
malate metabolic process	-	27	-
organic phosphonate transport	-	27	-
organophosphate ester transport	-	27	-
DNA duplex unwinding	-	26	-
RNA polyadenylation	-	26	-
negative regulation of kinase activity	-	26	-
negative regulation of protein kinase activity	-	26	-
chondroitin sulfate proteoglycan biosynthetic process	-	26	-
chloroplast RNA processing	-	25	-
RNA-dependent DNA replication	-	25	-
mRNA 3'-end processing	-	25	-
protein import into nucleus, docking	-	25	-
triterpenoid biosynthetic process	-	25	-
pyrimidine nucleoside monophosphate biosynthetic process	-	25	-
triterpenoid metabolic process	-	25	-
glycosaminoglycan catabolic process	-	25	-
iron ion homeostasis	-	24	-
base-excision repair	-	24	-
intracellular protein transmembrane transport	-	24	-
peptidyl-tyrosine dephosphorylation	-	24	-
basipetal auxin transport	-	24	-
positive regulation of GTPase activity	-	24	-
protein deacetylation	-	23	-
translational termination	-	23	-
pentacyclic triterpenoid metabolic process	-	22	-
pentacyclic triterpenoid biosynthetic process	-	22	-
pyrimidine ribonucleoside monophosphate metabolic process	-	22	-
purine ribonucleoside biosynthetic process	-	22	-
monovalent inorganic cation homeostasis	-	21	-
chloride transport	-	21	-
mismatch repair	32	21	-

histone deacetylation	-	21	-

* A dash mark indicates that no unigene is found in that subcategory.
