# Supplementary material for: De Novo Transcriptome Sequence Assembly from Coconut Leaves and Seeds with a Focus on Factors Involved in RNA-Directed DNA Methylation
Source: G3 (Bethesda). 2014 Sep 4;4(11):2147–57. doi: 10.1534/g3.114.013409 (PMC4232540; doi:10.1534/g3.114.013409)
Supplement: Supporting Information [file supp_g3.114.013409_TableS8.pdf]

Table S8 List of complete GO terms for biological process at level 8

Pathway	embryo	endosperm	leaf
Alanine, aspartate and glutamate metabolism	70	92	93
alpha-Linolenic acid metabolism	43	55	73
Amino sugar and nucleotide sugar metabolism	116	193	158
Aminoacyl-tRNA biosynthesis	76	102	102
Aminobenzoate degradation	53	72	82
Anthocyanin biosynthesis	5	2	9
Arachidonic acid metabolism	33	41	50
Arginine and proline metabolism	113	133	122
Ascorbate and aldarate metabolism	48	66	57
Atrazine degradation	5	4	4
Benzoate degradation	11	30	23
beta-Alanine metabolism	51	68	60
beta-Lactam resistance	1	3	1
Betalain biosynthesis	2	2	2
Biosynthesis of ansamycins	5	9	5
Biosynthesis of siderophore group nonribosomal peptides	0	3	3
Biosynthesis of terpenoids and steroids	1	0	1
Biosynthesis of unsaturated fatty acids	35	59	48
Biosynthesis of vancomycin group antibiotics	3	7	6
Biotin metabolism	18	28	28
Butanoate metabolism	36	53	54
Butirosin and neomycin biosynthesis	5	12	5
C5-Branched dibasic acid metabolism	13	19	20
Caffeine metabolism	9	12	27
Caprolactam degradation	13	22	15
Carbon fixation in photosynthetic organisms	93	140	142
Carbon fixation pathways in prokaryotes	65	104	102
Carotenoid biosynthesis	12	11	13
Chloroalkane and chloroalkene degradation	33	29	23
Chlorocyclohexane and chlorobenzene degradation	6	9	8
Citrate cycle (TCA cycle)	71	105	116
Cutin, suberine and wax biosynthesis	6	4	8
Cyanoamino acid metabolism	27	37	52
Cysteine and methionine metabolism	97	112	112
D-Alanine metabolism	6	4	4
D-Arginine and D-ornithine metabolism	3	2	3

D-Glutamine and D-glutamate metabolism	5	8	4
Diterpenoid biosynthesis	9	5	3
Drug metabolism - cytochrome P450	39	36	46
Drug metabolism - other enzymes	72	92	91
Ether lipid metabolism	34	42	36
Ethylbenzene degradation	3	6	5
Fatty acid biosynthesis	37	68	53
Fatty acid elongation	17	35	25
Fatty acid metabolism	70	94	93
Flavone and flavonol biosynthesis	5	5	13
Flavonoid biosynthesis	31	32	71
Fluorobenzoate degradation	4	8	7
Folate biosynthesis	12	14	14
Fructose and mannose metabolism	65	103	99
Galactose metabolism	89	108	111
Geraniol degradation	18	30	23
Glucosinolate biosynthesis	4	3	5
Glutathione metabolism	92	95	114
Glycerolipid metabolism	130	132	130
Glycerophospholipid metabolism	100	136	110
Glycine, serine and threonine metabolism	90	124	126
Glycolysis Gluconeogenesis	155	245	205
Glycosaminoglycan biosynthesis - chondroitin sulfate dermatan sulfate	22	26	22
Glycosaminoglycan biosynthesis - heparan sulfate heparin	33	39	32
Glycosaminoglycan biosynthesis - keratan sulfate	2	1	0
Glycosaminoglycan degradation	23	25	19
Glycosphingolipid biosynthesis - ganglio series	22	24	17
Glycosphingolipid biosynthesis - globo series	17	18	14
Glycosphingolipid biosynthesis - lacto and neolacto series	4	7	2
Glycosylphosphatidylinositol(GPI)-anchor biosynthesis	3	2	2
Glyoxylate and dicarboxylate metabolism	89	129	154
Histidine metabolism	28	31	27
Indole alkaloid biosynthesis	8	10	15
Inositol phosphate metabolism	86	97	71
Insect hormone biosynthesis	0	0	1
Isoflavonoid biosynthesis	0	2	5
Isoquinoline alkaloid biosynthesis	24	38	31
Limonene and pinene degradation	24	36	26

Linoleic acid metabolism	19	24	54
Lipoic acid metabolism	8	12	9
Lipopolysaccharide biosynthesis	5	11	9
Lysine biosynthesis	21	24	25
Lysine degradation	58	88	61
Metabolism of xenobiotics by cytochrome P450	37	38	49
Methane metabolism	101	240	183
Monoterpenoid biosynthesis	1	5	6
mTOR signaling pathway	17	18	14
Mucin type O-Glycan biosynthesis	2	1	0
Naphthalene degradation	13	7	7
N-Glycan biosynthesis	29	35	27
Nicotinate and nicotinamide metabolism	24	17	30
Nitrogen metabolism	112	190	183
Novobiocin biosynthesis	19	25	17
One carbon pool by folate	32	36	42
Other glycan degradation	52	45	40
Other types of O-glycan biosynthesis	7	7	10
Oxidative phosphorylation	94	163	149
Pantothenate and CoA biosynthesis	35	39	41
Penicillin and cephalosporin biosynthesis	2	4	3
Pentose and glucuronate interconversions	64	101	62
Pentose phosphate pathway	74	114	130
Peptidoglycan biosynthesis	10	31	5
Phenylalanine metabolism	100	139	114
Phenylalanine, tyrosine and tryptophan biosynthesis	69	84	77
Phenylpropanoid biosynthesis	53	77	84
Phosphatidylinositol signaling system	109	107	74
Phosphonate and phosphinate metabolism	2	9	5
Photosynthesis	3	5	3
Polyketide sugar unit biosynthesis	7	11	10
Porphyrin and chlorophyll metabolism	65	73	87
Primary bile acid biosynthesis	5	13	5
Propanoate metabolism	55	83	75
Purine metabolism	351	415	412
Pyrimidine metabolism	148	197	172
Pyruvate metabolism	107	162	170
Retinol metabolism	22	22	37

Riboflavin metabolism	31	44	45
Selenocompound metabolism	36	38	37
Sesquiterpenoid and triterpenoid biosynthesis	1	0	1
Sphingolipid metabolism	72	70	63
Starch and sucrose metabolism	274	409	272
Steroid biosynthesis	19	37	34
Steroid degradation	7	6	8
Steroid hormone biosynthesis	11	32	40
Stilbenoid, diarylheptanoid and gingerol biosynthesis	4	6	10
Streptomycin biosynthesis	18	47	38
Styrene degradation	17	18	19
Sulfur metabolism	50	64	68
Synthesis and degradation of ketone bodies	8	10	14
T cell receptor signaling pathway	120	137	118
Taurine and hypotaurine metabolism	7	9	8
Terpenoid backbone biosynthesis	33	52	57
Tetracycline biosynthesis	9	14	12
Thiamine metabolism	79	78	81
Toluene degradation	11	19	17
Tropane, piperidine and pyridine alkaloid biosynthesis	28	42	29
Tryptophan metabolism	71	88	87
Tyrosine metabolism	53	68	52
Ubiquinone and other terpenoid-quinone biosynthesis	23	31	31
Valine, leucine and isoleucine biosynthesis	19	25	22
Valine, leucine and isoleucine degradation	66	87	87
Various types of N-glycan biosynthesis	22	26	20
Vitamin B6 metabolism	8	21	17
Xylene degradation	3	1	2
Zeatin biosynthesis	4	6	8
**Total**	**5686**	**7707**	**7231**
